# Phosphoproteomics Reveals Regulation of Secondary Metabolites in *Mahonia bealei* Exposed to Ultraviolet-B Radiation

**DOI:** 10.3389/fpls.2021.794906

**Published:** 2022-01-11

**Authors:** Amin Liu, Shengzhi Liu, Yaohan Li, Minglei Tao, Haote Han, Zhuoheng Zhong, Wei Zhu, Jingkui Tian

**Affiliations:** ^1^The Cancer Hospital of the University of Chinese Academy of Sciences (Zhejiang Cancer Hospital), Institute of Basic Medicine and Cancer, Chinese Academy of Sciences, Hangzhou, China; ^2^College of Biomedical Engineering and Instrument Science, Zhejiang University, Hangzhou, China

**Keywords:** phosphoproteomics, *Mahonia bealei*, ultraviolet-B radiation, abiotic stress, secondary metabolism

## Abstract

Mahonia bealei (M. bealei) is a traditional Chinese medicine containing a high alkaloid content used to treat various diseases. Generally, only dried root and stem are used as medicines, considering that the alkaloid content in *M. bealei* leaves is lower than in the stems and roots. Some previous research found that alkaloid and flavonoid contents in the *M. bealei* leaves may increase when exposed to ultraviolet B (UV-B) radiation. However, the underlying mechanism of action is still unclear. In this study, we used titanium dioxide material enrichment and mass-based label-free quantitative proteomics techniques to explore the effect and mechanism of *M. bealei* leaves when exposed to UV-B treatment. Our data suggest that UV-B radiation increases the ATP content, photosynthetic pigment content, and some enzymatic/nonenzymatic indicators in the leaves of *M. bealei*. Moreover, phosphoproteomics suggests phosphoproteins related to mitogen-activated protein kinase (MAPK) signal transduction and the plant hormone brassinosteroid signaling pathway as well as phosphoproteins related to photosynthesis, glycolysis, the tricarboxylic acid cycle, and the amino acid synthesis/metabolism pathway are all affected by UV-B radiation. These results suggest that the UV-B radiation activates the oxidative stress response, MAPK signal transduction pathway, and photosynthetic energy metabolism pathway, which may lead to the accumulation of secondary metabolites in *M. bealei* leaves.

## Introduction

*Mahonia Bealei* is a kind of evergreen shrub widely grown in China and some other countries in Asia, Europe, and America. Its main active ingredients are berberine, jatrorrhizine, and palmatine, which have heat-clearing, detoxifying, and antiphlogistic effects and may be used to treat stomach disorders, inflammation, infections like hepatitis, and skin-related diseases. In addition, a previous study (Deng et al., 2013) points out that, besides berberine, jatrorrhizine, and palmatine, Alishan *Mahonia* contains columbamine, and its 1-diphenyl-2-trinitrophenylhydrazine (DPPH) radical scavenging activity is five times higher than the activity of vitamin C. Alishan *Mahonia* may exert a certain inhibitory effect on pain (Hu et al., 2011). Moreover, studies also find that jateorhizine, berberine, and palmatine from *Mahonia* can inhibit the activity of fungus (Wang et al., 2018). So far, only dried roots and stems named *Gonglaomu* were officially used as medicines in China, considering that the alkaloid contents in *M. bealei* leaves are lower than those in stems and roots (Zhu et al., 2015). Therefore, studies on increasing the alkaloid contents in *M. bealei* leaves could be valuable for improving medicinal material utilization.

As one of the environmental factors, UV-B radiation can alter the primary and secondary metabolism in plants. Studies show that UV-B stimulation can significantly increase the content of phenols and the activity of antioxidant enzymes in *Salvia Miltiorrhiza* leaves (Liu et al., 2013). Wang et al. (2016) find that UV-B radiation can stimulate tanshinone production in hairy root cultures of *Salvia Miltiorrhiza*, and when combining UV-B radiation with methyl jasmonate treatment, the content of tanshinone can be increased by 4.9 times over the control. Moreover, an integrative omic study indicates that the NAC1 gene is positively involved in the increase of salvianolic acid in *S. Miltiorrhiza* under UV-B radiation (Ying et al., 2020). Chen et al. (2020) investigated the metabolic variations in *Dendrobium officinal Kimura et Migo* stem under UV-B radiation and found that the treatment may increase flavonoids, polysaccharides, and alkaloids. Furthermore, in *Chrysanthemum morifolium* leaves, caffeoylquinic acid, fatty acid, and flavonoid contents all increased when the leaves were exposed to UV-B radiation (Yang et al., 2018). In previous studies, we also find that the contents of benzylisoquinoline alkaloids, such as berberine, jateorhizine, palmatine, and columbamine, in *M. bealei* leaves increased under UV-B radiation and dark treatment (Zhang L. et al., 2014; Zhu et al., 2021). We also discovered that the enhanced tricarboxylic acid (TCA) cycle under UV-B radiation might regulate the energy metabolism to provide extra power for the biosynthesis of secondary metabolites. However, the mechanism of induction is not fully understood.

Protein post-translational modifications (PTMs) include protein phosphorylation, ubiquitylation, glycosylation, methylation, acetylation, and so on. Protein phosphorylation is one of the most well-studied modification types involving the regulation of transcription, translation, protein degradation, and cell signaling and communication. For instance, sucrose nondependent protein kinase 2.10 (SnRK2.10) protein phosphorylation in *Arabidopsis thaliana* under salt stress is regarded as the response to dehydration pressure (Maszkowska et al., 2019), and phosphorylation increased in proteins related to secondary metabolism in sugarcane under drought stress (Vital et al., 2017). In *C. roseus*, UV-B radiation activates calcium-dependent protein phosphorylation, the glycolysis pathway, and the TCA-cycle proteins phosphorylation changes as well as an oxidative stress response affecting secondary metabolic pathways, such as aromatic amino acids and phenylpropanoids (Zhong et al., 2019). Yet the protein phosphorylation levels in *M. bealei* under UV-B radiation might be related to metabolic changes, especially for alkaloids.

In this study, we used titanium dioxide (TiO_2_) material enrichment and mass-based label-free quantitative proteomics techniques to explore the effect and mechanism of *M. bealei* leaves when exposed to UV-B treatment. Label-free proteomic and mass spectrum detection techniques were applied for phosphopeptide identification. Furthermore, phosphoproteomic results were confirmed by qRT-PCR analyses.

## Materials and Methods

### Plant Material and Treatment

Seedlings of *Mahonia bealei* (Fort.) Carr. were provided by the College of Pharmacy Zhejiang University (Hangzhou, China). Plants were 3 years old and grown in the greenhouse. UV-B treatment was then carried out in special UV-B chambers with four UV-B lamps (Philips 20 W ultraviolet B TL20W/12RS, Philips). The intensity of UV-B irradiation on the leaf surface was 104.4 kJ m ^–2^ d ^–1^, which was measured from 275 to 320 nm with a UV radiometer (Beijing Normal University photoelectric instrument factory, Beijing, China). The following conditions were applied: 26°C and humidity of 58%. The plants were divided into two groups: plants exposed to UV-B radiation for 6 h were labeled as the treatment or UV-B group and the control group comprised those growing under regular light for 6 h. After the parallel treatment, the leaves were collected and snap-frozen in liquid nitrogen and then stored in a refrigerator at −80°C for subsequent analysis. Three independent biological replicates were conducted in our study (Supplementary Figure 1).

### Measurement of ATP Content

ATP-content determination was carried out using an ATP Content Assay kit (Solarbio, Beijing, China). In simple terms, fresh leaves of *M. bealei* were fully ground on ice with 1 mL extraction buffer and then centrifuged by 8,000 × g at 4°C for 10 min. The supernatant was collected and mixed with 500 μL of chloroform, followed by centrifugation of 10,000 × g at 4°C for 3 min, after which the supernatant was collected. The water bath reaction was performed according to the requirements of the kit. The ATP content was obtained by comparing the absorbance increase at 340 nm before and after the reaction with the absorbance increase of ATP standard substance.

### Analysis of Physiological Changes

The peroxidase activity (POD), superoxide dismutase activity (SOD), total antioxidant capacity (T-AOC), plant malondialdehyde (MDA) content, and hydrogen peroxide (H_2_O_2_) content were measured by different kits following the manufacturer’s protocols (Nanjingjiancheng, Jiangsu, China). Phenylalanine ammonia-lyase (PAL) activity was determined as previously described (Huang et al., 2010) with minor modifications. Leaf samples were homogenized within 1 mL of extracting solution on ice and centrifuged by 12,000 × g at 4°C for 30 min. The supernatant was used in the final reaction mixture. PAL activity was measured by recording the absorbance at 290 nm.

Contents of carotenoids (Car), chlorophyll a (Chl a), and chlorophyll b (Chl b) were determined according to Wellburn and Lichtenthaler (1984). The content of brassinosteroid was determined by a Plant Brassinosteroids (BR) ELISA Kit (Boshen Biotechnology, Jiangsu, China).

For the total flavonoid assay, 0.5 mL of 5% NaNO_2_ was added into 1 mL methanol extraction of leaves. Next, 0.5 mL of 10% Al (NO_3_)_3_ was added to the reaction mixture and mixed with 1.5 mL of 2 M NaOH. After incubation for 15 min, the absorbance was measured at 510 nm (Li et al., 2015). Total anthocyanin content was measured as described by Neff and Chory (1998). For total alkaloid analysis, the obtained residue was dissolved in 1 mL of phosphate buffer solution (pH 4.5) after which the solution was transferred to a glass tube, and 1 mL of bromocresol green solution (0.03%) was added. After 30 min, 1 mL of chloroform was added, followed by vortexing for 2 min. The lower layer was separated after 5 min. The extraction was repeated once, and the lower phase was combined. The extracts were analyzed by using a UV–Vis spectrophotometer at 418 nm. The data were expressed as mg berberine equivalents (BE)/g fresh weight (FW) with berberine as a reference standard for the total alkaloid assay (Zhu et al., 2021). Next, the Pearson correlation coefficient was used to analyze the correlation between BR, total alkaloids, total flavonoids, and anthocyanins.

### Protein Extraction

A portion (0.5 g) of the samples were ground into powder in liquid nitrogen and then transferred to acetone solution containing 10% trichloroacetic acid and 0.07% 2-mercaptoethanol. After vortexing, the samples were incubated at −20°C for 1 h, and the supernatant was discarded after centrifugation at 9,000 × g for 20 min at 4°C. The precipitation was then cleaned twice with acetone solution containing 0.07% 2-mercaptoethanol. After drying, the precipitation was lysed at room temperature for 1 h with lysis buffer (7 M urea, 2 M thiourea, 5% CHAPS, and 2 mM tributylphosphine). The resulting suspension was centrifuged at 20,000 × g for 20 min at 25°C, the supernatant was collected as total protein, and the protein concentration was determined by the Bradford method with bovine serum albumin as the standard.

### Protein Purification and Digestion

One hundred microgram proteins were adjusted to the final volume of 100 μL. Next, 400 μL of methanol, 100 μL of chloroform, and 300 μL of double distilled water were added successively. After mixing, the sample protein was centrifuged at 20,000 × g for 10 min. The upper phase was discarded, and 300 μL of methanol was added to the lower phase and centrifuged at 20,000 × g for 10 min again. Solid particles were collected for subsequent enzymatic hydrolysis.

The purified protein was suspended in 50 mM ammonium bicarbonate, then reduced with 50 mM dithiothreitol at 56°C for 30 min in the dark, and subsequently alkylated with 50 mM iodoacetamide at 37°C for 30 min in the dark. Finally, the proteins were digested with trypsin at a 1:100 enzyme/protein ratio at 37°C for 16 h in the dark and preliminary desalted with Strata X Column (Phenomenex, United States).

### Phosphopeptide Enrichment

The phosphorylated peptide enrichment kit (PTM BIO, Hangzhou, China) was used to enrich phosphopeptides. Peptides (1 mg) were resuspended in 1 mL of binding buffer. After mixing, 50 μL of TiO_2_ materials (8 mg) were added and lightly shaken for 30 min. They were centrifuged by 2,000 × g for 3 min to discard the supernatant, and TiO_2_ materials were washed using washing buffer. Finally, phosphopeptides were eluted, dried, and dissolved in 0.1% formic acid for nanoflow liquid chromatography-mass spectrometry.

### Analysis Using Nanoliquid Chromatography-Mass Spectrometry

The peptides dissolved in 0.1% (v/v) formic acid were subjected to a homemade reverse analysis column (15 cm length, 75 μm i.d.). Buffer phase A was the 0.1% formic acid and 2% acetonitrile, and buffer phase B contained 90% acetonitrile and 0.1% formic acid. On an EASY nLC 1000 UPLC System, the following linear separation gradient was set: 0–86 min, 17% B. 86–110 min, 17–28% B, 110–115 min, 28–80% B, 115–120 min at 80% B, flow rate 500 nL/min.

The peptides were subjected to an NSI source followed by tandem mass spectrometry (MS/MS) in Q Exactive™ Plus (Thermo) coupled online to the UPLC. The full scanning m/z scan range was 350–1,800 with a voltage of 2.0 kV. An Orbitrap detects the complete peptide at 70,000 resolution. For selecting the peptides for MS/MS, HCD was set to 28% to detect fragments at 35,000 resolution in the Orbitrap A data-dependent program that scans 20,000 ions/s after one MS scan, dynamically excludes 15.0 s. Automatic gain control (AGC) was set to 1E5. The fixed first mass was set to 100 m/z.

### Protein Identification

Maxquant (V1.5.2.8) was used to retrieve the secondary mass spectrometry data and set the retrieval parameters: identified proteins were searched against the UniProtKB database (uniprot-reviewed viridiplantae with 40,254 entries, downloaded 2020.04.22). A reverse database was added to calculate the false positive rate (FPR) caused by random matching, and a common pollution database was added to the database to eliminate the influence of contaminated proteins in the identification results. Trypsin was used for digestion. The number of missing bits was set to two. The first search and the main search level mother ion quality error tolerance were set to 20 and 5 ppm, respectively. The quality of secondary ions had an error tolerance of 0.02 Da. The cysteine alkylation was set to fixed, variable modifiers for oxidation of methionine, protein N acetylation, deamidation (NQ), and protein tyrosine phosphorylation of serine-threonine identification PSM of FDR was set to 1%.

### Functional Analysis of Phosphoproteins

The submitted proteins were annotated for subcellular localization using Wolfpsort,^1^ a software that predicts subcellular localization. Heatmap Illustrator (heml.1.0.3.7) was used to provide an overall representation of intensities of the phosphorylation site of only phosphorylated proteins in the control or UV-B group. MapMan bin codes were used to categorize phosphoproteins. DAVID functional annotation analysis has phosphoproteins that were classified into molecular function, cellular component, and the biological process by GO terms. Then, the results were presented by the micro-bioinformation platform.^2^ The Kyoto Encyclopedia of Genes and Genomes (KEGG) database^3^ was used to annotate protein pathways, and the pathways were enriched by Metascape^4^ and performed by the micro-bioinformation platform. Phosphoproteins’ protein--protein interactions were analyzed in STRING.^5^ The required confidence score was set as >0.700 for highly confident interactions. A K-means algorithm in STRING clusters the network.

### Motif Analysis of Phosphorylation Modification Sites

The Motif-X algorithm was used to extract significantly enriched amino acid motifs surrounding the identified phosphosites. The sequence window was limited to 13 amino acids, and foreground peptides were prealigned with the phosphosite centered. The proteome data set from the Uniprot database were used as the background database. The occurrence threshold was set at the minimum of 20 peptides, and the *P*-value threshold was set at < 10^–6^.

### RNA Extraction and Quantitative Real-Time PCR

About 0.1 g of fresh leaf samples were taken from *M. bealei* and ground into powder in a sterilized mortar with liquid nitrogen. RNA was extracted from leaf tissue using a Quick RNA Isolation kit (Huayueyang Biotechnology, Beijing, China). After that, the sample RNA was reverse transcribed using a Reverse Transcription System (5 × All-In-OneRT MasterMix, abmgood, Canada). Primers were designed using the Primer Premier 5.0, and qRT-PCR was performed with MasterMiX-No Dyekit on a CFX real-time PCR detection system (Bio-rad, Hercules, CA, United States). Finally, quantitative differences between groups were evaluated by the relative quantitative method (2^–ΔΔ*CT*^). Actin was used as a single reference gene (Zhu et al., 2019).

## Results

### ATP-Content Change in *Mahonia bealei* Leaves Exposed to Ultraviolet B Radiation

**FIGURE 1:**
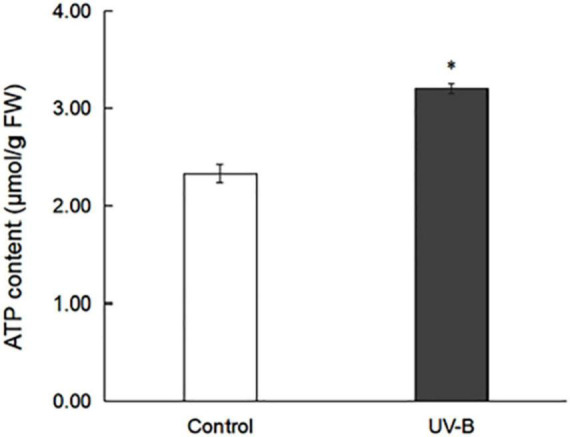
ATP content in *M. bealei* leaves exposed to UV-B radiation. ATP content was determined by analyzing the increase in absorbance at 340 nm before and after the reaction. ATP content was calculated as μmol per fresh weight (g). Data are shown as means ± SD from three independent biological replicates. Student’s *t*-test was used to compare values between the control and UV-B groups. *p < 0.05.

ATP from the control and UV-B groups in *M. bealei* leaves was extracted and analyzed. The results show that ATP contents in the control and UV-B groups were 2.33 and 3.20 μmol g^–1^, respectively (Figure 1 and Supplementary Table 1), which indicates that UV-B radiation increased the ATP content of *M. bealei* leaves. Because ATP can provide phosphate groups for the phosphorylation process, this pre-experiment result also reflects the difference in phosphorylation levels between groups to some extent (Zhong et al., 2019).

### Physiological and Chemical Changes in *Mahonia bealei* Leaves Exposed to Ultraviolet B Radiation

**FIGURE 2:**
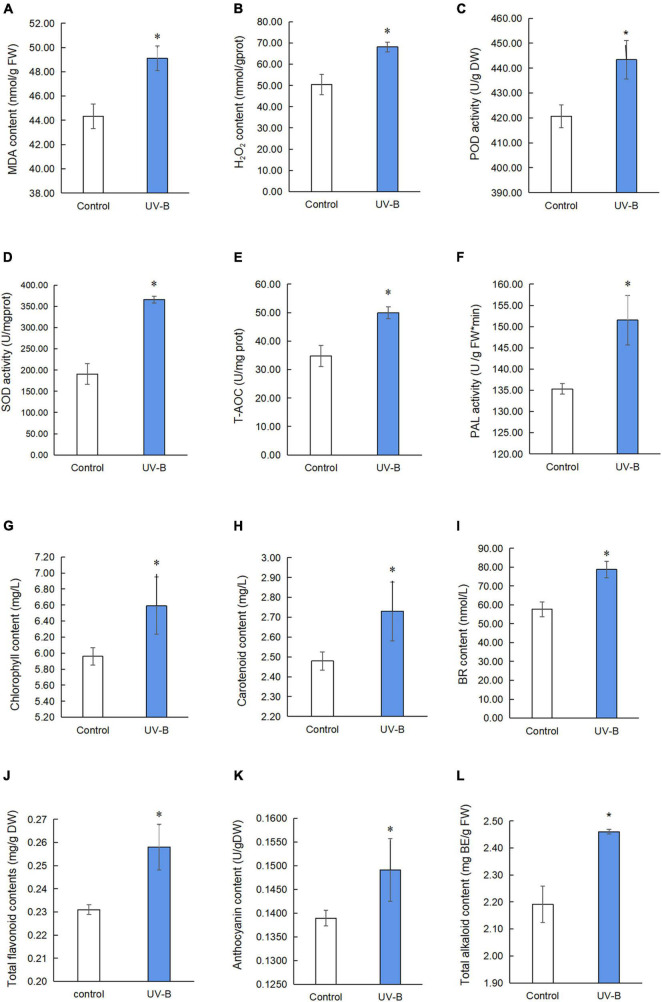
Physiological and chemical analyses of *M. bealei* leaves exposed to UV-B radiation. **(A)** MDA content; **(B)** hydrogen peroxide (H_2_O_2_); **(C)** POD activity; **(D)** SOD activity; **(E)** T-AOC; **(F)** PAL activity; **(G)** chlorophyll content; **(H)** carotenoid content; **(I)** BR content; **(J)** total flavonoid contents; **(K)** anthocyanin content; **(L)** total alkaloids content. Data are presented as mean ± SD from three independent biological replicates. POD, peroxidase; SOD, superoxide dismutase; T-AOC, total antioxidant capacity; MDA, malondialdehyde; PAL, phenylalanine ammonia-lyase; BR, brassinosteroid; DW, dry weight; FW, fresh weight; UV-B, ultraviolet B treatment for 6 h. *p < 0.05.

To study oxidative stress, MDA content, H_2_O_2_ content, POD activity, SOD activity, T-AOC, and PAL activity in *M. bealei* leaves exposed to UV-B radiation were determined (Figures 2A–F and Supplementary Table 1). The results show that MDA and H_2_O_2_ contents were increased, and these antioxidant enzyme activities were significantly increased after UV-B radiation. The contents of photosynthetic pigments (chlorophyll and carotenoids) were also increased after UV-B radiation (Figures 2G,H and Supplementary Table 1).

Next, the content of brassinosteroid, a plant hormone, was determined by an ELISA kit. The results showed that the content of brassinosteroids in *M. bealei* leaves was significantly increased after UV-B induction (Figure 2I and Supplementary Table 1). In addition, secondary metabolites, such as total flavonoid, anthocyanin, and total alkaloid content, were also increased (Figures 2J–L and Supplementary Table 1). Moreover, the correlation analysis of BR, total alkaloids, total flavonoids, and anthocyanins showed that BR had a significant correlation with total alkaloids and total flavonoids (Supplementary Figure 2).

### Identification and Functional Categories of Phosphoproteins in *Mahonia bealei* Leaves Exposed to Ultraviolet B Radiation

**FIGURE 3:**
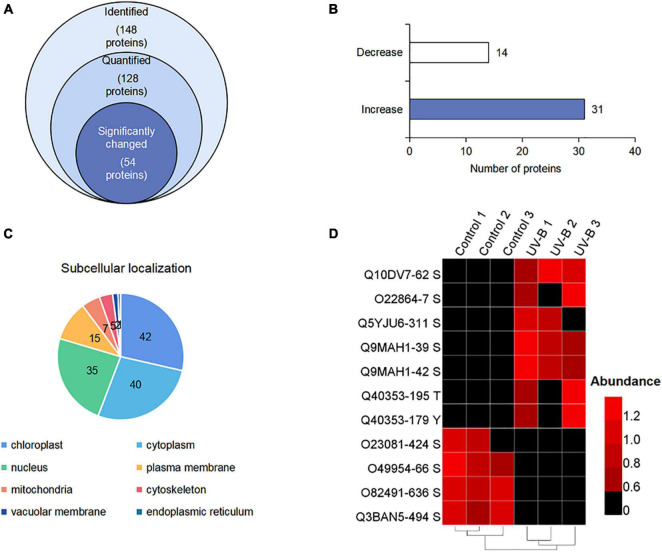
(Phospho) proteome response upon UV-B stress in *M. bealei* leaves. **(A)** Venn diagram of all identified phosphoproteins; **(B)** bar chart of significantly changed phosphoproteins; **(C)** subcellular localization analysis of phosphorylated proteins; **(D)** heat map showing values of phosphosite intensities of phosphorylated and absolutely dephosphorylated proteins under UV-B radiation.

To display the phosphorylation situation of proteins in *M. bealei* under UV-B radiation, phosphoproteomic analysis was performed (Supplementary Figure 2). One hundred ninety-two phosphorylation sites on 148 proteins were identified, of which 128 proteins were successfully quantified (Supplementary Table 2). The data were used for the subsequently differential site analysis; 54 significantly changed phosphoproteins were identified in the UV-B group, of which 31 and 14 phosphoproteins were increased and decreased, respectively (*p* < 0.05, fold change > 1.2) (Figures 3A,B). Subcellular localization of the identified phosphorylated proteins shows that phosphorylated proteins are mainly located in chloroplast, cytoplasm, and nucleus (Figure 3C). In addition, nine proteins were phosphorylated only in the control or UV-B groups (Figure 3D).

MapMan bin codes were used for functional category analysis of phosphoproteins. One hundred forty-eight phosphoproteins were classified into 24 categories. They were mainly related to protein synthesis/post-translational modification/degradation, signaling, RNA transcriptional regulation, cell organization/vesicle transport, and photosynthesis (Supplementary Figure 4). GO term classification was involved in protein phosphorylation and photosynthesis of biological process (BP); ATP binding and protein binding of cellular component (CC); plasma membrane and cytoplasm of molecular function (MF) (Supplementary Figure 5).

**FIGURE 4:**
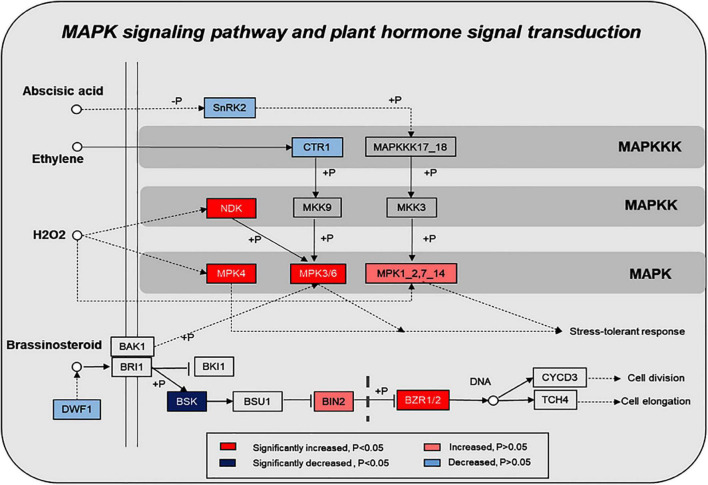
Changes of proteins in plant hormone signal transduction and MAPK signaling pathway in response to the UV-B treatments. The pathways are drawn based on the KEGG database. SNRK2, serine/threonine-protein kinase SRK2; CTR, serine/threonine-protein kinase CTR1; NDK, nucleoside-diphosphate kinase; MPK: mitogen-activated protein kinase; DWF1, Delta24-sterol reductase; BSK, BR-signaling kinase; BIN2, protein brassinosteroid insensitive 2; BZR1/2, brassinosteroid resistant 1/2.

**FIGURE 5:**
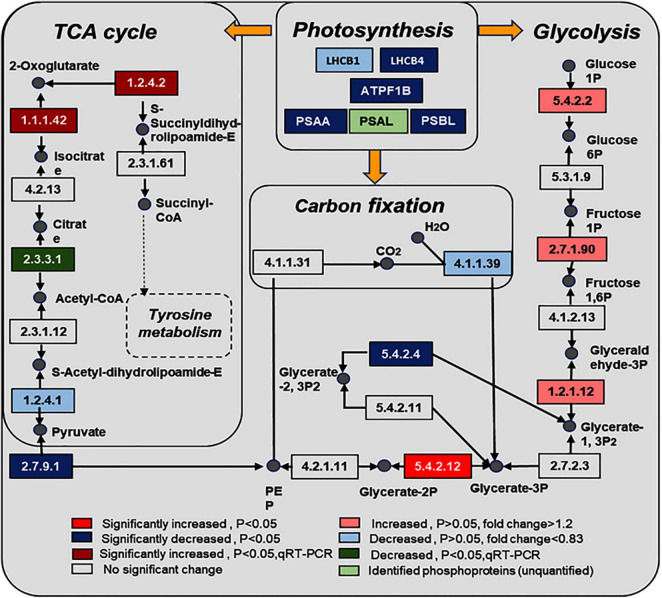
Changes of proteins in photosynthesis, glycolysis, carbon fixation, and citrate cycle in response to the UV-B treatments. The pathways are drawn based on the KEGG database. LHCB1, light-harvesting complex II chlorophyll a/b binding protein 1; LHCB4, light-harvesting complex II chlorophyll a/b binding protein 4; PSAA, photosystem I P700 chlorophyll a apoprotein A1; PSAL, photosystem I subunit XI; PSBL, photosystem II PsbL protein; ATPF1B, F-type H^+^/Na^+^-transporting ATPase subunit beta; EC numbers: 5.4.2.2, phosphoglucomutase (PGM); 2.7.1.90, diphosphate dependent phosphofructokinase; 1.2.1.12, glyceraldehyde 3-phosphate dehydrogenase (GAPDH); 5.4.2.12:2, 3-bisphosphoglycerate-independent phosphoglycerate mutase; 2.7.9.1, pyruvate, orthophosphate dikinase (PPSD); 1.2.4.1, pyruvate dehydrogenase E1 component (OSJ); 1.1.1.42, isocitrate dehydrogenase (CICDH); 1.2.4.2, 2-oxoglutarate dehydrogenase E1 component (odhA); 4.1.1.39, ribulose-bisphosphate carboxylase large chain; 5.4.2.4, bisphosphoglycerate/phosphoglycerate mutase; 2.3.3.1, citrate synthase (ACLA).

**FIGURE 6:**
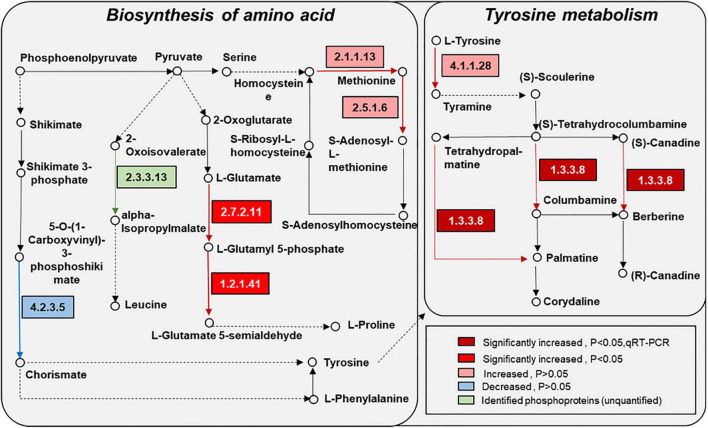
Changes of proteins in amino acid synthesis and metabolism in response to the UV-B treatments. The pathways are drawn based on the KEGG database. EC numbers: 4.2.3.5, chorismate synthase; 2.3.3.13, 2-isopropylmalate synthase; 2.1.1.13, 5-methyltetrahydropteroyltriglutamate-homocysteine methyltransferase; 2.7.2.11, glutamate 5-kinase; 1.2.1.41, glutamate-5-semialdehyde dehydrogenase; 2.5.1.6, S-adenosylmethionine synthetase; 4.1.1.28, aromatic-L-amino-acid/L-tryptophan decarboxylase; 1.3.3.8, tetrahydroprotoberberine oxidase (STOX).

KEGG pathway enrichment found that phosphoproteins were mainly involved in MAPK signaling pathways, photosynthesis, glycolysis, plant hormone signal transduction, and biosynthesis of amino acids. Moreover, many phosphoproteins were also involved in the secondary metabolic pathways (Supplementary Figure 6). Combined with KEGG pathway enrichment results, KEGG mapping and KEGG color were used to match the differentially phosphorylated proteins into the KEGG pathway. Using qRT-PCR verification results, three pathways were obtained through integrated analysis: MAPK signal pathway and plant hormone signaling (Figure 4), photosynthesis-glycolysis-TCA cycle pathway (Figure 5), and amino acid biosynthesis pathway (Figure 6).

### Motif Analysis of Protein Phosphorylation Modifications

**FIGURE 7:**
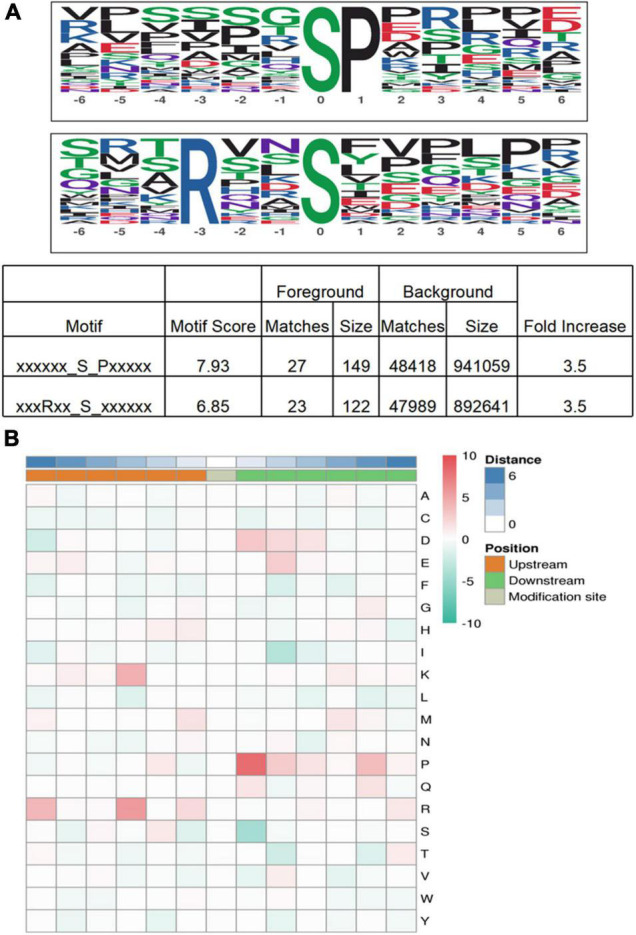
Motif analysis of phosphoproteins in *M. bealei* leaves exposed to UV-B radiation. **(A)** This modified site feature sequence and its enrichment statistics are from MoMo software and Motif-X analysis for overrepresented phosphorylation motifs of all identified phosphosites in *M. bealei* leaves; **(B)** The motif enrichment heat map of upstream and downstream amino acids of all identified modification sites. Red indicates that this amino acid is significantly enriched near the modification site, and green indicates that this amino acid is significantly reduced near the modification site.

Protein motif analysis calculated the trend of amino acid sequences in the phosphorylation site region by counting the regularity of amino acid sequences before and after all phosphorylation sites in the sample. Motif-X analysis results revealed that, for S-phosphorylation, the [SP], were potential substrates of MAPK, cyclin-dependent kinase, and cyclin-dependent kinase-like, and that [RxxS], which can be identified by mitogen-activated protein kinase kinase (MAPKK), calmodulin-dependent protein kinase (CaMK)II, and protein kinase A, were the most enriched motif (Wang K. et al., 2014; Zhang et al., 2014; Figure 7A).

The motif enrichment heat map of upstream and downstream amino acids of all identified modification sites shows that arginine (R) and proline (P) near the serine residue (S) were the most conserved amino acids upstream and downstream of modified serine sites, and arginine (R) and proline (P) were significantly overrepresented at positions 3 and −1, respectively. In addition, lysine (K) frequently occurred at position 3, whereas aspartic acid (D) and glutamic acid (E) were overrepresented at positions −1 and −2, respectively (Figure 7B).

### Protein–Protein Interaction Analysis of Differential Phosphoproteins

Protein–protein interaction networks of the phosphoproteins were obtained using the open-access STRING software. One hundred forty-two nodes connected by 127 interactions in phosphoproteins, 51 nodes connected by 19 interactions in significantly changed phosphoproteins were found. Cluster networks analysis in the STRING software showed that most compacts were constituted by phosphoproteins involved in carbon metabolism, ribosome, photosynthesis, oxidative phosphorylation, brassinosteroid, and MAPK cascade pathways (Supplementary Figure 7A). Most compacts were constituted by significantly changed phosphoproteins involved in ribosome, photosynthesis, and oxidative phosphorylation pathways (Supplementary Figure 7B).

### Quantitative Real-Time PCR Analyses

**FIGURE 8:**
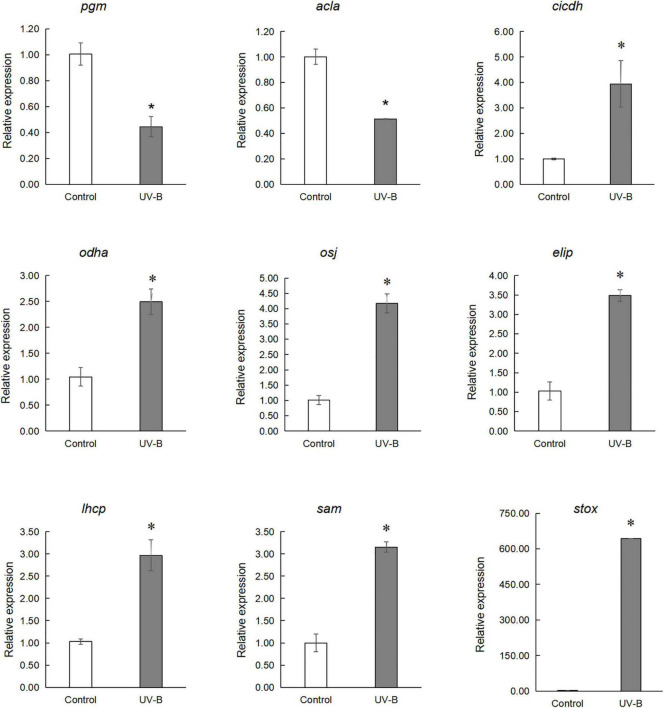
qRT-PCR analysis of genes involved in the light reaction, glycolysis, TCA cycling, and secondary metabolism. Early light-induced protein 2 (*elip*), chlorophyll a-b binding protein 4.1 (*lhcb*), cytosolic isocitrate dehydrogenase (*cicdh*), 2-oxoglutarate dehydrogenase E1/E2 component (*odha*), (S)-tetrahydroprotoberberine oxidase (*stox*), pyruvate dehydrogenase E1 component subunit alpha (*osj*) and S-adenosylmethionine synthase 1 (*sam*) expression increased in UV-B group, but quantitative results of 2,3-bisphosphoglycerate-independent phosphoglycerate mutase (*pgm*) and Citrate synthase (*acla*) are reduced in UV-B group (*p < 0.05).

qRT-PCR analysis of several genes involved in the light reaction, glycolysis, TCA cycle, and secondary metabolism was performed. Early light-induced protein 2 (*elip*), chlorophyll a-b binding protein4.1 (*lhcb*), cytosolic isocitrate dehydrogenase (*cicdh*), 2-oxoglutarate dehydrogenase E1/E2 component (*odha*), (s)-tetrahydroprotoberberine oxidase (*stox*), pyruvate dehydrogenase E1 component subunit alpha (*osj*) and s-adenosylmethionine synthase1 (*sam*) expression increased in the UV-B group, but quantitative results of 2,3-bisphosphoglycerate-independent phosphoglycerate mutase (*pgm*) and citrate synthase (*acla*) were reduced compared to the control group (Figure 8 and Supplementary Table 3).

## Discussion

### Oxidative Stress Induces Secondary Metabolite Biosynthesis in *Mahonia bealei* Leaves Exposed to Ultraviolet B Radiation

UV-B radiation can induce oxidative stress, which produces reactive oxygen species (Ortega-Hernández et al., 2019) and then activates the plant ROS scavenging system (Das and Roychoudhury, 2014). Our study results show that the contents of MDA and H_2_O_2_ in *M. bealei* leaves were increased after exposure to UV-B radiation, and correspondingly, the activity of POD and SOD and T-AOC under the UV-B radiation was significantly improved (*p* < 0.05), which was significant for the detoxification of oxidants produced by UV-B (Czégény et al., 2016; Köhler et al., 2017). Kasote et al. (2015) suggest that the compounds involved in the phenylpropanoid pathway and the phenolics are important for antioxidation, which is consistent with our results. In the present study, PAL activity, total flavonoids, and anthocyanin contents were significantly increased in *M. bealei* leaves exposed to UV-B radiation (Figures 2F,J,K). UV-B radiation promotes the accumulation of flavonoids and anthocyanins, which can also be interpreted as a way for plants to prevent cell damage. Previous studies show that flavonoids and anthocyanins, as UV-B absorbing compounds and antioxidants, have a significant role in reducing the adverse effect of UV radiation on plant cells (Julkunen-Tiitto et al., 2015; Su et al., 2016). This study suggests that the oxidative damage of *M. bealei* leaves could be alleviated by the accumulation of flavonoids and anthocyanins under UV-B radiation.

### Ultraviolet B Induces Secondary-Metabolite Biosynthesis by Activating the Mitogen-Activated Protein Kinase Signal Pathway and Regulating Brassinosteroids

Functional categorization of identified phosphoproteins indicates that these proteins are mainly involved in protein metabolism, signaling, RNA, cell, and photosynthesis. Furthermore, the pathway enrichment analysis shows that MAPK signaling and plant hormone signaling transduction pathways are significantly enriched in response to UV-B radiation. The identified phosphoproteins are mapped into the KEGG database; the results show that eight and six phosphoproteins were significantly changed in the plant MAPK signaling pathway and plant hormone signaling transduction pathway, respectively (Figure 4).

MAPK cascades are the common feature in eukaryotic cells. As a general feature, there are three kinases, including MAP3K (Mitogen-Activated Protein Kinase Kinase Kinase, MAPKKK), MAP2K (Mitogen-activated protein kinase, MAPKK), and MAPK. Among them, MAPK cascades are important for plant growth (Zhang et al., 2018), development (Chen et al., 2018; Sun et al., 2018; Xue et al., 2020), and abiotic stress (de Zelicourt et al., 2016; Ding et al., 2018). Moreover, MAPK cascades are more likely involved in secondary metabolisms, such as regulation of camalexin (Thulasi Devendrakumar et al., 2018), nicotine (De Boer et al., 2011), anthocyanin (Lei et al., 2014), and phytoalexin biosynthesis (Genot et al., 2017). Recent studies show that phytohormones also participated in the regulation of secondary metabolite biosynthesis under environmental stresses in plants (Chu et al., 2011; Yang et al., 2012; Li et al., 2017). In this study, KEGG pathway analysis shows that some phosphorylated proteins, such as BR-signaling kinase (BSK), brassinosteroids resistant 1/2 (BZR1/2), and protein brassinosteroid insensitive 2 (BIN2), are associated with the brassinosteroid regulation signaling pathway, and the phosphorylated protein Delta24-sterol reductase (DWF1) is associated with brassinosteroid biosynthetic changes after exposure to UV-B induction. Therefore, we speculated that UV-B radiation activates the brassinosteroid biosynthesis pathway and the related signal transduction. Next, we determined the content of brassinosteroid in the samples, and our results show that UV-B stimulates brassinosteroid content, which was significantly increased in *M. bealei* leaves (Figure 2J). In *Arabidopsis thaliana*, the BR signal from the cell membrane receptor BRI1 (brassinosteroid insensitive 1) passed through the phosphorylation-mediated cascade BSK1-BSU1-BIN2/GSK1 (BSK1—BRI1 suppressor 1—BIN2/glycogen synthase kinase-1), eventually reaching the transcription factor BZR1, which can trigger BR response (Wang et al., 2012, 2014). Some studies show that inhibition of BR biosynthesis may weaken plant photosynthesis and affect plant tolerance to stress (Cui et al., 2011; Jiang et al., 2012; Ahammed et al., 2014). Also, Cui et al. (2012) suggest that BR utilizes hydrogen peroxide (H_2_O_2_)- and nitric oxide (NO)-mediated mechanisms to provide stress tolerance. Additionally, brassinosteroids have a certain role in stimulating secondary metabolic synthesis in plants. For example, Ahammed et al. (2012) find that brassinosteroids stimulate the accumulation of flavonoids in tomato leaves. Similarly, Xi et al. (2013) find that the applications of BR can promote anthocyanin biosynthesis in grapes.

We conducted a simple correlation statistical analysis on the contents of BR, total alkaloids, total flavonoids, and anthocyanins. Our results show that BR had a significant correlation with total alkaloids and total flavonoids (Supplementary Figure 2). Furthermore, crosstalk was observed in the MAPK signaling pathway and brassinosteroid signaling pathway: BR could reduce the activation of BIN2 to inhibit mitogen-activated protein kinase kinase 4, 5, 7, and 9 (MKK4/5/7/9), which induce the phosphorylation of mitogen-activated protein kinase 3 and 6 (MPK3/6) in negatively regulated stomatal cell formation (Kim et al., 2012). Therefore, our data suggest that the MAPK signal pathway and plant hormone signal transduction pathway, especially the BR signaling pathway, are activated by UV-B radiation and can affect the synthesis mechanism of subsequent secondary metabolites.

### Ultraviolet Radiation Stimulates the Combined Action Mechanism of Phosphoproteins in the Photosynthetic–Glycolysis–Tricarboxylic Acid Cycle Pathway

Phosphoprotein KEGG pathway enrichment analysis shows that carbon metabolism–related pathways were significantly enriched, such as photosynthesis, glycolysis, TCA cycle, and carbon fixation, and proteins involved in these pathways were significantly changed. The phosphorylation level of light-harvesting complex II chlorophyll a/b binding protein 4 (LHCB4), photosystem I P700 chlorophyll an apoprotein A1 (PSAA), and photosystem II PsbL protein (PSBL) were significantly decreased in photosynthesis. At the same time, phosphorylated 2,3-bisphosphoglycerate-independent phosphoglycerate mutase was upregulated, and bisphosphoglycerate/phosphoglycerate mutase was downregulated in glycolysis. Studies show that UV-B radiation can affect membrane-binding proteins, such as enzymes involved in pigment synthesis, and lead to degradation damage of plant thylakoid membranes (Pradhan et al., 2006). Our experimental results show that the contents of chlorophyll and carotenoids were significantly increased, which may be related to photosynthesis’s function of photosynthetic pigments in absorbing and transferring light energy or causing primary photochemical reactions. Glycolysis and the TCA cycle were the fundamental pathways to produce energy for plant survival, and the phosphorylation level of proteins related to glycolysis and TCA pathways were significantly changed. Furthermore, the ATP content in the leaves under UV-B radiation was significantly increased (Figure 1). We know that photosynthesis and secondary metabolites are intrinsically related (Maeda, 2019), and secondary metabolites were induced by UV-B radiation to absorb and/or dissipate solar energy, which might alleviate the UV-B damage to plants (Liu et al., 2012). These results suggest that UV-B radiation, which stimulates the combined action mechanism of phosphoproteins in the photosynthetic–glycolysis–TCA cycle pathway, may consequently provide the substrate and energy for the synthesis of secondary metabolites.

### Amino Acid Metabolism Was Enhanced by Ultraviolet B Radiation to Provide the Precursors for Flavonoid and Alkaloid Biosynthesis

Pathway enrichment analysis showed that the biosynthesis of the amino acid pathway was significantly enriched. Also, the identified phosphoproteins were mainly involved in amino acid biosynthesis and metabolism (Figure 6). Phosphorylated delta-1-pyrroline-5-carboxylate synthetase, which is involved in the proline synthesis pathway, was significantly increased in the UV-B group compared with the control group. Phosphorylated chorismate synthase involved in the biosynthesis of tyrosine and phenylalanine and 5-methyltetrahydropteroyltri glutamate-homocysteine methyltransferase and s-adenosylmethionine synthetase involved in the methionine biosynthesis were also altered. Amino acids and their derivatives have an important role in protein synthesis, growth and development, and stress of plants (Hildebrandt et al., 2015). Moreover, the metabolism of amino acids is closely related to energy metabolism, carbon and nitrogen balance, and secondary metabolism (Pratelli and Pilot, 2014). Specifically, plant amino acids, such as phenylalanine, tyrosine, and tryptophan, help protein synthesis and affect the biosynthesis of many growth hormones and secondary metabolites, which often could resist biotic and abiotic stress (Tzin and Galili, 2010). In this study, the contents of total flavonoid and anthocyanin in *M. bealei* leaves were increased under UV-B radiation because of the enhanced PAL activity, which may result from changes of phosphorylated proteins in phenylalanine biosynthesis and metabolism-related pathways. Tyrosine was the central hub to myriad specialized metabolic pathways, and phenylalanine and tyrosine were also the initial substrates for isoquinoline alkaloid biosynthesis. The phosphorylated aromatic L-amino acid/L-tryptophan decarboxylase, which was involved in the preliminary metabolism of tyrosine, was upregulated. Moreover, phosphorylated PAL, a key enzyme in the flavonoid biosynthesis pathway, and phosphorylated S-adenosylmethionine synthetase, an important enzyme in the alkaloid biosynthesis pathway, also significantly changed. Additionally, gene expression of *stox* and *sam*, which were involved in the biosynthesis of the berberine, columbamine, and palmatine, were significantly upregulated. Previous literature also shows that UV-B induced total flavones and alkaloids’ biosynthesis (Zhu et al., 2021), which is consistent with our data. In addition, the total alkaloid content in samples of the control and UV-B treatment groups was verified in this study, and it was found that the alkaloid content in leaves of the UV-B group was increased (Figure 2L). Accordingly, we conclude that the amino acid biosynthesis and metabolic pathways activated by UV-B are induced to promote some secondary metabolites’ biosynthesis in *M. bealei* leaves.

## Conclusion

In the present study, we analyze the phosphoproteomics of *M. bealei* leaves that were exposed to UV-B radiation for 6 h. The results show that ultraviolet-B radiation stimulates the oxidative stress system, MAPK signaling, amino acid synthesis, and metabolism pathway leaves and promotes the accumulation of secondary metabolites, total flavonoids, and total alkaloids in *M. bealei*. The analysis of phosphoproteins in these pathways led to the following conclusions: (1) oxidative stress induces secondary metabolite biosynthesis in *M. bealei* leaves under UV-B radiation; (2) UV-B induces secondary metabolite biosynthesis by activating the MAPK signal pathway and regulating brassinosteroid (BR); (3) UV-B radiation stimulates the combined action mechanism of phosphoproteins in the photosynthetic–glycolysis–TCA cycle pathway; (4) UV-B-activates amino acid synthesis and the metabolism pathway in leaves promoting the synthesis of secondary metabolites flavonoid and alkaloid. This study provides a new perspective for exploring the ultraviolet-induced mechanism of increasing alkaloid content of the secondary metabolites of *M. bealei* leaves. However, due to the imperfection of the transcriptome database and insufficient abundance of the phosphoproteomics data, this mechanism needs to be further analyzed.

## Data Availability Statement

The data presented in the study are deposited in the ProteomeXchange repository, accession number PXD028873.

## Author Contributions

AL and WZ conceived the idea, designed the research, and drafted the manuscript. AL performed the experiments and analyzed the data. SL, ZZ, and JT supervised the study. MT, YL, and HH provided suggestions for the revision of the manuscript. All authors read and approved the final manuscript.

## Conflict of Interest

The authors declare that the research was conducted in the absence of any commercial or financial relationships that could be construed as a potential conflict of interest.

## Publisher’s Note

All claims expressed in this article are solely those of the authors and do not necessarily represent those of their affiliated organizations, or those of the publisher, the editors and the reviewers. Any product that may be evaluated in this article, or claim that may be made by its manufacturer, is not guaranteed or endorsed by the publisher.

## Footnotes

1
http://www.genscript.com/psort/wolf_psort.html

2
http://www.bioinformatics.com.cn

3
http://www.kegg.jp/

4
http://metascape.org/gp/

5
http://string-db.org/

## Supplementary Material

Supplementary Figure 1 Map of the experimental design process.

Supplementary Figure 2 Correlation analysis of brassinosteroid with total alkaloids and flavonoids of secondary metabolites.

Supplementary Figure 3 The quality control analysis. **(A)** Pearson’s correlation coefficient. **(B)** The relative standard deviation (RSD). **(C)** Three methods of statistical analysis to evaluate quantitative repeatability.

Supplementary Figure 4 Functional categories of phosphoproteins in *M. bealei* leaves exposed to UV-B radiation. The functional category of phosphoproteins was determined using MapMan bin codes. One hundred forty-eight phosphoproteins were mapped and classified into 24 categories. The top four categories are presented in detail with pie charts. “Not assigned” indicates protein without ontology.

Supplementary Figure 5 The top classified enriched GO terms involved in UV-B radiation.

Supplementary Figure 6 The KEGG pathway enrichment bubble map of phosphoproteins under UV-B induced. KEGG pathway analysis shows that phosphoproteins are mainly involved in MAPK signaling pathways, photosynthesis, glycolysis, plant hormone signal transduction, and biosynthesis of amino acids. Moreover, many phosphoproteins have been identified in the secondary metabolic pathways.

Supplementary Figure 7 Protein–protein interaction analysis of identified phosphoproteins in *M. bealei* leaves under UV-B radiation.
