# Supplementary figures and images for: Phosphoproteomics Reveals Regulation of Secondary Metabolites in Mahonia bealei Exposed to Ultraviolet-B Radiation
Source: Front Plant Sci. 2022 Jan 11;12:794906. doi: 10.3389/fpls.2021.794906 (PMC8787227; doi:10.3389/fpls.2021.794906)

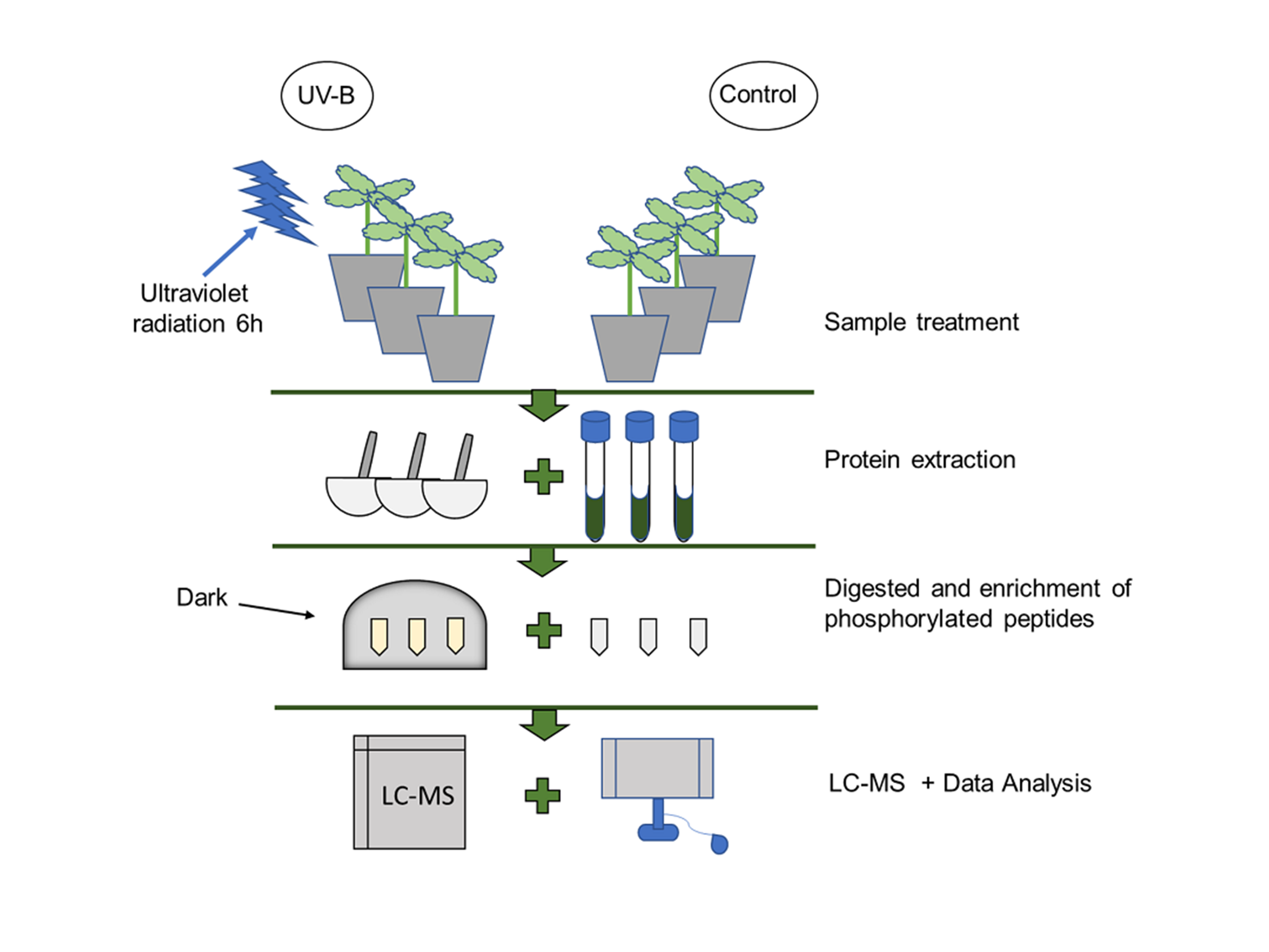

Supplement: Supplementary Figure 1 — Map of the experimental design process. [file Image_1.TIF]

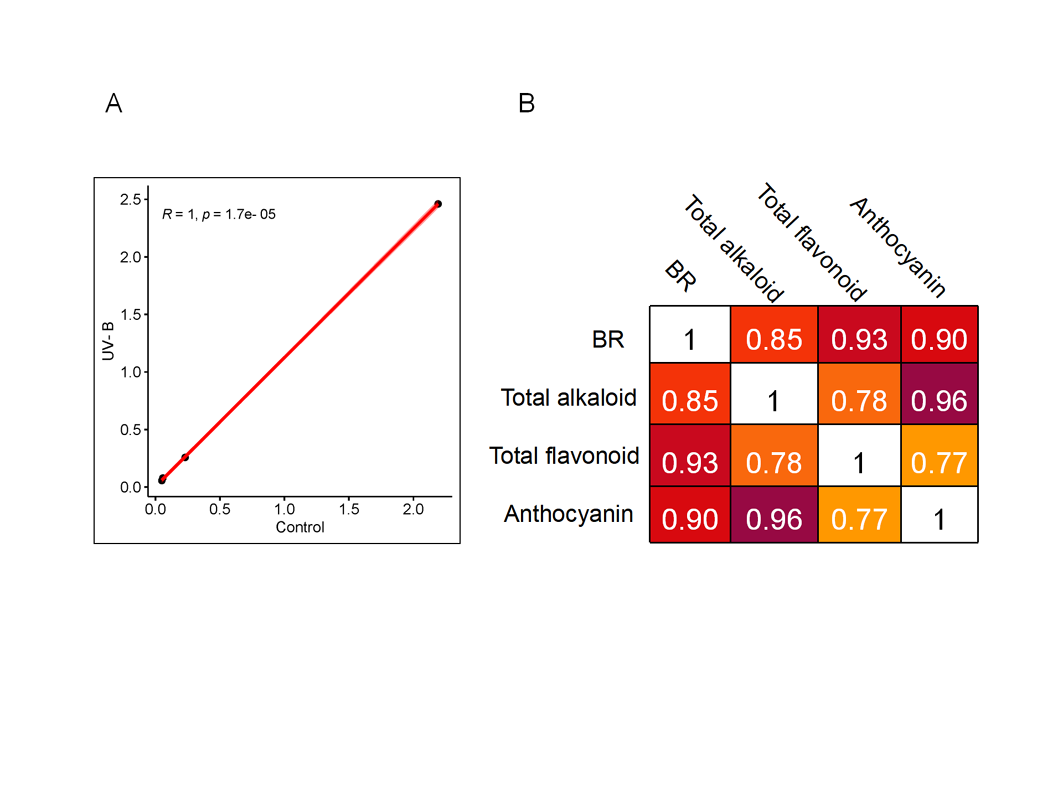

Supplement: Supplementary Figure 2 — Correlation analysis of brassinosteroid with total alkaloids and flavonoids of secondary metabolites. [file Image_2.TIF]

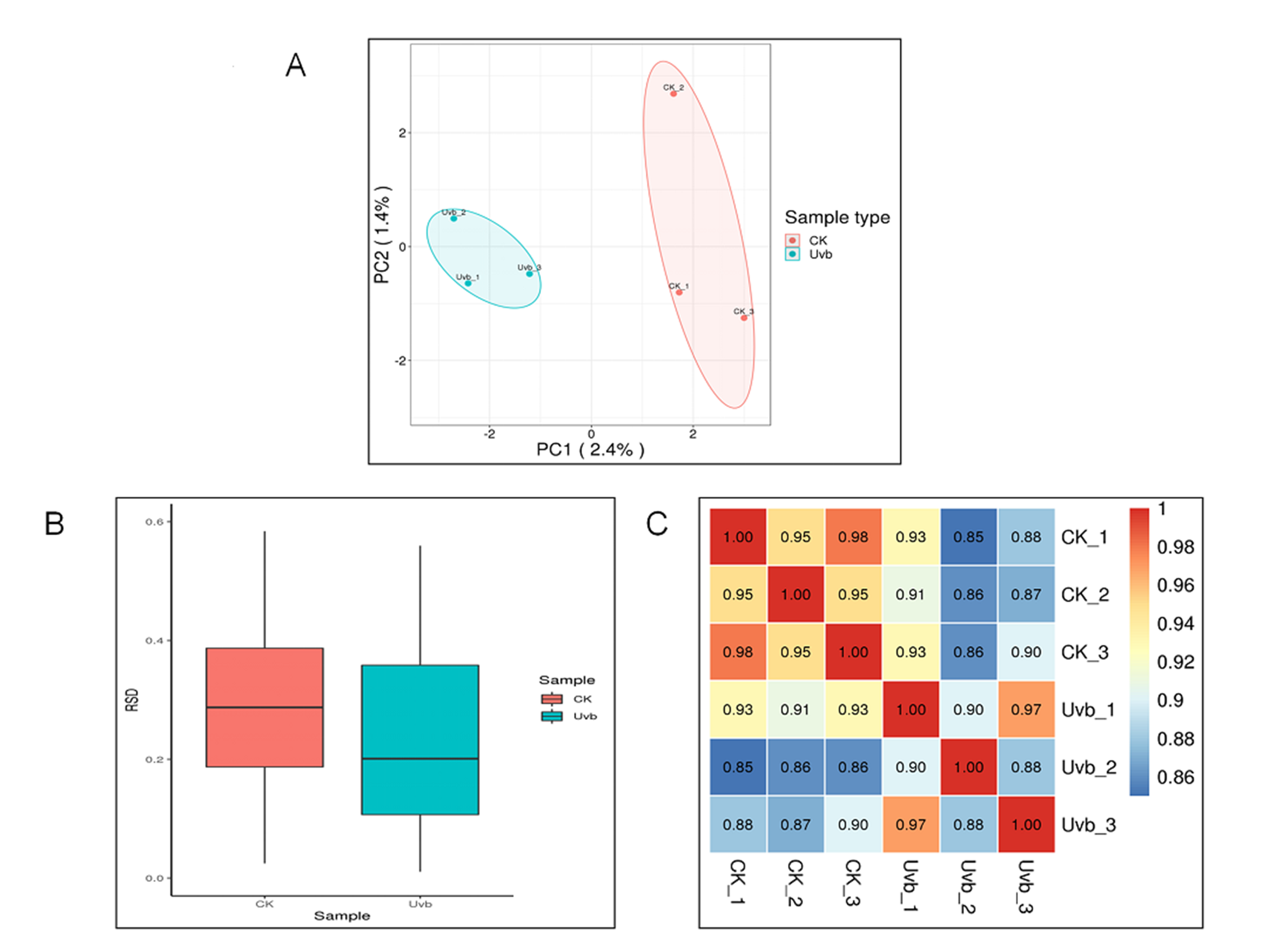

Supplement: Supplementary Figure 3 — The quality control analysis. (A) Pearson’s correlation coefficient. (B) The relative standard deviation (RSD). (C) Three methods of statistical analysis to evaluate quantitative repeatability. [file Image_3.TIF]

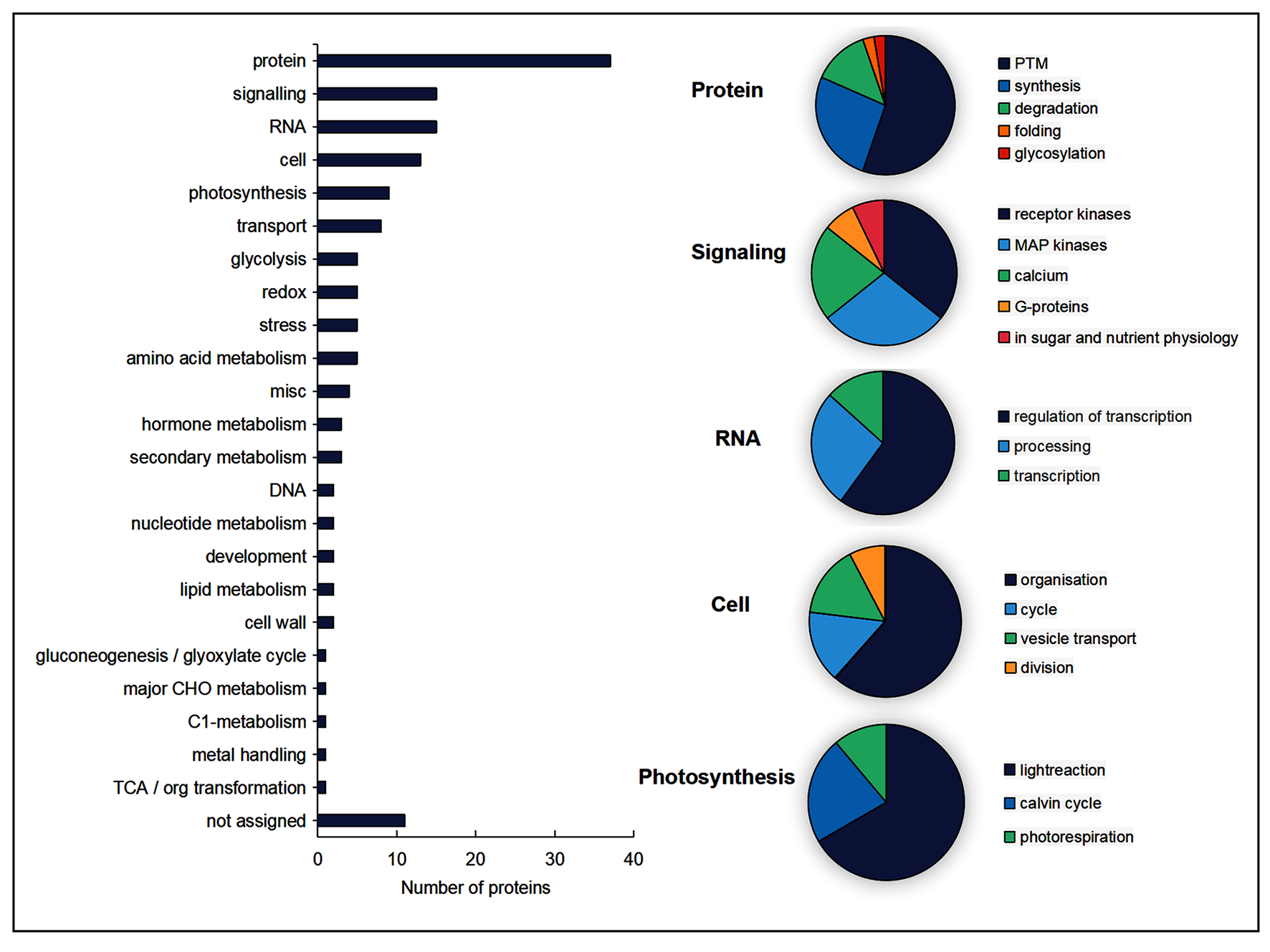

Supplement: Supplementary Figure 4 — Functional categories of phosphoproteins in M. bealei leaves exposed to UV-B radiation. The functional category of phosphoproteins was determined using MapMan bin codes. One hundred forty-eight phosphoproteins were mapped and classified into 24 categories. The top four categories are presented in detail with pie charts. “Not assigned” indicates protein without ontology. [file Image_4.TIF]

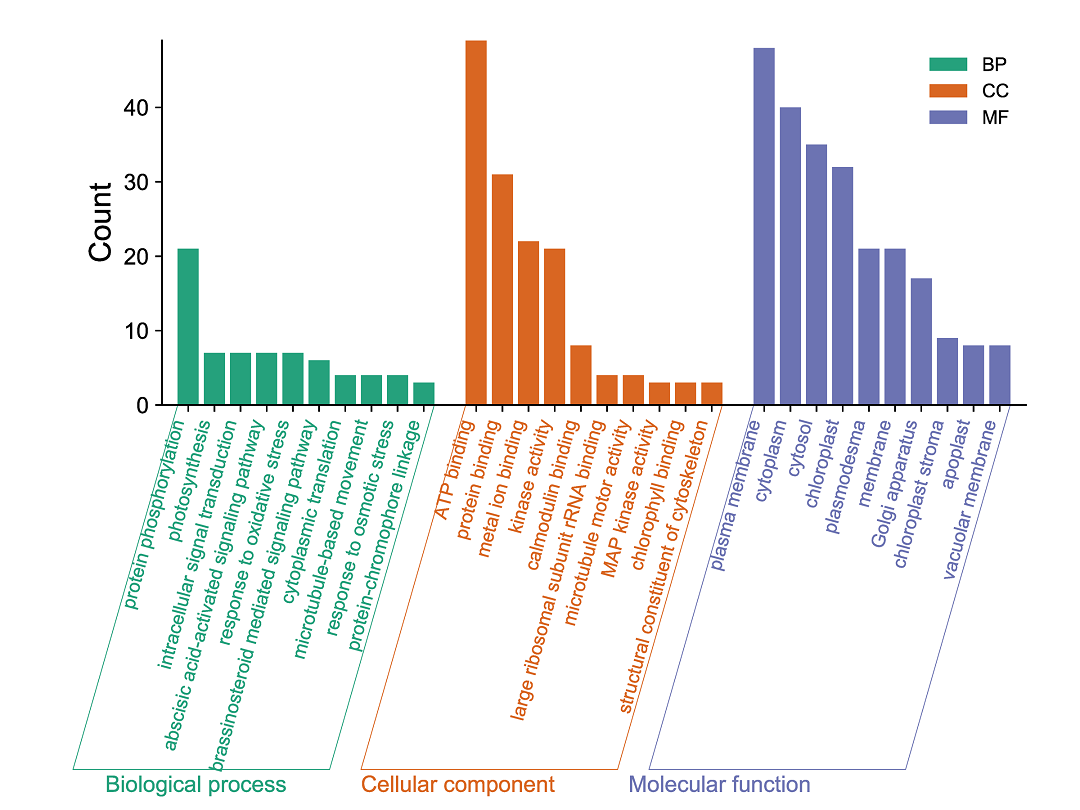

Supplement: Supplementary Figure 5 — The top classified enriched GO terms involved in UV-B radiation. [file Image_5.TIF]

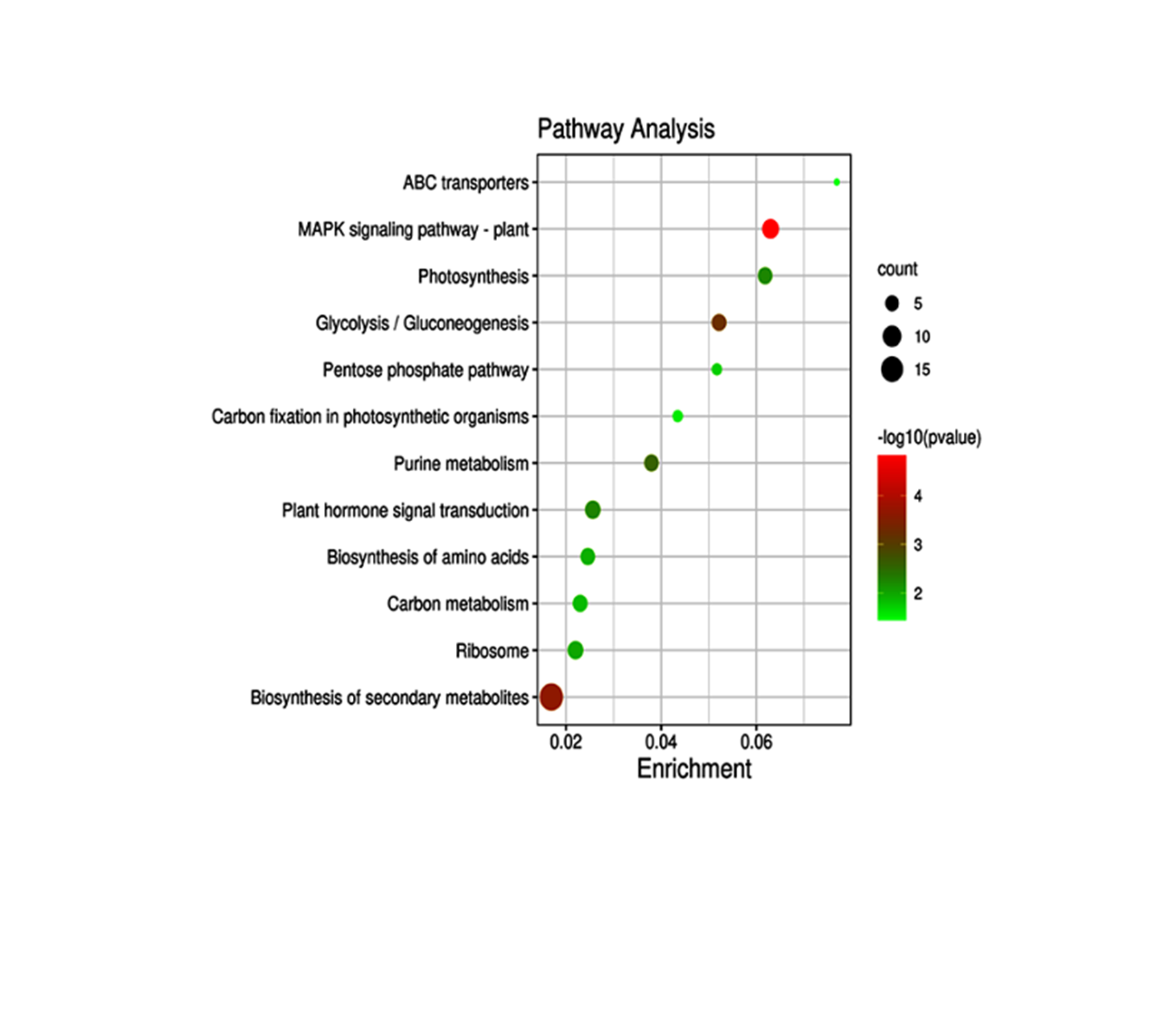

Supplement: Supplementary Figure 6 — The KEGG pathway enrichment bubble map of phosphoproteins under UV-B induced. KEGG pathway analysis shows that phosphoproteins are mainly involved in MAPK signaling pathways, photosynthesis, glycolysis, plant hormone signal transduction, and biosynthesis of amino acids. Moreover, many phosphoproteins have been identified in the secondary metabolic pathways. [file Image_6.TIF]

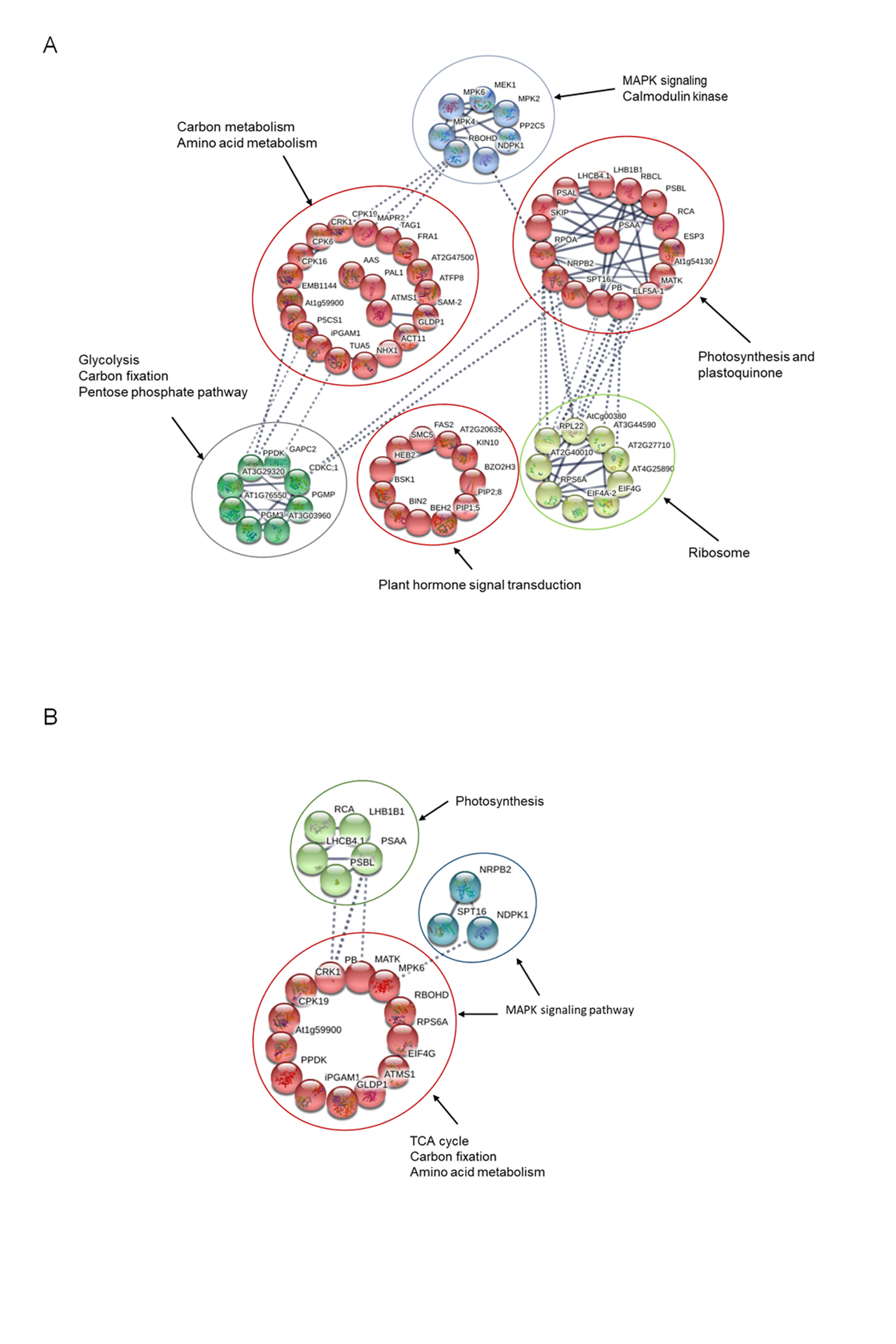

Supplement: Supplementary Figure 7 — Protein–protein interaction analysis of identified phosphoproteins in M. bealei leaves under UV-B radiation. [file Image_7.TIF]
