# Supplementary material for: Phosphoproteomics Reveals Regulation of Secondary Metabolites in Mahonia bealei Exposed to Ultraviolet-B Radiation
Source: Front Plant Sci. 2022 Jan 11;12:794906. doi: 10.3389/fpls.2021.794906 (PMC8787227; doi:10.3389/fpls.2021.794906)
Supplement: Supplementary file 8 [file Table_1.DOCX]

**Table S1.** Physiological and chemical changes in *M. bealei* leaves exposed to UV-B treatment

| **Group** | ATP content  (mol/g FW） | | POD activity  (U/g DW) | SOD activity (U/mg prot） | T-AOC  (U/mg prot) | | PAL activity  (U /g FW*min) | |
| --- | --- | --- | --- | --- | --- | --- | --- | --- |
| **Control** | 2.33±0.0958 | | 420.72±4.6096 | 190.25±24.4298 | 34.79±3.7219 | | 135.33±1.2496 | |
| **UV-B** | 3.20±0.0524 | | 443.36±7.7504 | 365.78±8.0512 | 49.3±2.0751 | | 151.51±5.8721 | |
| **Group** | | MDA content (nmol/g FW) | H_2_O_2_ content （mmol/gprot) | Chlorophyll content (mg/L) | | Carotenoid content (mg/L) | | BR content (nmol/L) |
| **Control** | | 44.33±1.1238 | 50.48±4.7681 | 5.96±0.1075 | | 2.48±0.0457 | | 57.64±3.9550 |
| **UV-B** | | 49.11±0.8559 | 68.12±2.2914 | 6.59±0.3568 | | 2.73±0.1486 | | 78.71±4.4495 |
| **Group** | | Total flavonoid contents  (mg/g DW) | Anthocyanin content（U/g DW） | Total alkaloid content (mg BE/g FW) | |  |  |  |
| **Control** | | 0.23±0.0021 | 0.1394±0.0017 | 2.19±0.0672 | |  |  |  |
| **UV-B** | | 0.26±0.0098 | 0.1491±0.0066 | 2.46±0.0085 | |  |  |  |
