# Supplementary material for: Phosphoproteomics Reveals Regulation of Secondary Metabolites in Mahonia bealei Exposed to Ultraviolet-B Radiation
Source: Front Plant Sci. 2022 Jan 11;12:794906. doi: 10.3389/fpls.2021.794906 (PMC8787227; doi:10.3389/fpls.2021.794906)
Supplement: Supplementary file 9 [file Table_2.DOCX]

**Table S2.** Identified phosphoproteins in M. bealei leaves exposed to UV-B radiation.

| **Protein ID^a^** | | **Description** | |  |
| --- | --- | --- | --- | --- |
| A0A068FIK2 | | Kinesin-like protein KIN-4A | |  |
| A0A0P0WY03 | | Diacylglycerol O-acyltransferase 1-2 | |  |
| Q8S8X4 | | Photosystem I P700 chlorophyll a apoprotein A1 | |  |
| Q5JN63 | | Cellulose synthase A catalytic subunit 4 [UDP-forming] | |  |
| Q6K5F8 | | Cyclin-dependent kinase G-1 | |  |
| Q6AT26 | | Probable cellulose synthase A catalytic subunit 1 [UDP-forming] | |  |
| Q53WK1 | | Glucosamine inositolphosphorylceramide transferase 1 | |  |
| F4I4F2 | | Serine/threonine-protein kinase RHS3 | |  |
| A9P822 | | S-adenosylmethionine synthase 1 | |  |
| P0DO09 | | Disease resistance protein Piks-1 | |  |
| B9FDE0 | | Probable serine/threonine-protein kinase BSK3 | |  |
| B9FFA3 | | Kinesin-like protein KIN-7E, chloroplastic | |  |
| B9FXV5 | | Eukaryotic translation initiation factor 4G | |  |
| C0LGN2 | | Probable leucine-rich repeat receptor-like serine/threonine-protein kinase At3g14840 | |  |
| F4HNU6 | | Nardilysin-like | |  |
| F4HU55 | | Serine/threonine-protein kinase BSK4 | |  |
| Q9LE81 | | Probable serine/threonine protein kinase IRE | |  |
| F4IL57 | | Kinesin-like protein KIN-14I | |  |
| F4IVI0 | | Mitotic checkpoint serine/threonine-protein kinase BUB1 | |  |
| F4JIF4 | | Protein NETWORKED 1B | |  |
| O23342 | | Preprotein translocase subunit SECE1 | |  |
| O24413 | | 60S acidic ribosomal protein P3 | |  |
| O24415 | | 60S acidic ribosomal protein P2B | |  |
| O48737 | | Thioredoxin M1, chloroplastic | |  |
| Q42662 | | 5-methyltetrahydropteroyltriglutamate--homocysteine methyltransferase | |  |
| O64639 | | Receptor-like serine/threonine-protein kinase At2g45590 | |  |
| O65361 | | Delta-1-pyrroline-5-carboxylate synthase | |  |
| O65570 | | Villin-4 | |  |
| Q6K8D9 | | SNW/SKI-interacting protein A | |  |
| Q10KY3 | | Calcium/calmodulin-dependent serine/threonine-protein kinase 1 | |  |
| P27490 | | Chlorophyll a-b binding protein 8, chloroplastic | |  |
| P08221 | | Chlorophyll a-b binding protein of LHCII type I, chloroplastic (Fragment) | |  |
| P0DSP5 | | Lectin | |  |
| P21342 | | Pyrophosphate--fructose 6-phosphate 1-phosphotransferase subunit alpha | |  |
| P26521 | | Glyceraldehyde-3-phosphate dehydrogenase, cytosolic | |  |
| P27067 | | Ribulose bisphosphate carboxylase large chain (Fragment) | |  |
| P45734 | | Phenylalanine ammonia-lyase | |  |
| P28567 | | Cell division control protein 2 homolog 2 (Fragment) | |  |
| P29345 | | 40S ribosomal protein S6 (Fragment) | |  |
| P37900 | | Heat shock 70 kDa protein, mitochondrial | |  |
| Q42877 | | DNA-directed RNA polymerase II subunit RPB2 | |  |
| P39207 | | Nucleoside diphosphate kinase 1 | |  |
| Q7XKA8 | | Serine/threonine-protein kinase SAPK5 | |  |
| P43293 | | Probable serine/threonine-protein kinase PBL11 | |  |
| P46252 | | 60S acidic ribosomal protein P2A | |  |
| P50346 | | 60S acidic ribosomal protein P0 | |  |
| Q6Z5N4 | | Pyruvate dehydrogenase E1 component subunit alpha-1, mitochondrial | |  |
| P53682 | | Calcium-dependent protein kinase 23 | |  |
| Q9AXQ6 | | Eukaryotic translation initiation factor 5A-1 | |  |
| P93262 | | Phosphoglucomutase, cytoplasmic | |  |
| P93472 | | Delta(24)-sterol reductase | |  |
| Q05609 | | Serine/threonine-protein kinase CTR1 | |  |
| Q0V869 | | OVARIAN TUMOR DOMAIN-containing deubiquitinating enzyme 11 | |  |
| Q1PFH8 | | Calcium-dependent protein kinase 19 | |  |
| Q2HXL0 | | Respiratory burst oxidase homolog protein C | |  |
| Q6Z0E2 | | Cation-chloride cotransporter 1 | |  |
| Q32026 | | Ribulose bisphosphate carboxylase large chain (Fragment) | |  |
| Q32RV5 | | 50S ribosomal protein L22, chloroplastic | |  |
| Q336V9 | | Serine/threonine-protein kinase BSK1-2 | |  |
| Q40977 | | Monodehydroascorbate reductase | |  |
| Q41870 | | Aquaporin PIP1-1 | |  |
| Q42450 | | Ribulose bisphosphate carboxylase/oxygenase activase B, chloroplastic | |  |
| Q42807 | | Stearoyl-[acyl-carrier-protein] 9-desaturase, chloroplastic | |  |
| Q42908 | | 2,3-bisphosphoglycerate-independent phosphoglycerate mutase | |  |
| Q68KI4 | | Sodium/hydrogen exchanger 1 | |  |
| Q5H7P5 | | Mannosylglycoprotein endo-beta-mannosidase | |  |
| Q6NPP4 | | Calmodulin-binding transcription activator 2 | |  |
| Q6ZCF0 | | Probable gamma-aminobutyrate transaminase 3, mitochondrial | |  |
| Q6ZH85 | | Plant intracellular Ras-group-related LRR protein 2 | |  |
| Q6ZKL8 | | Probable protein phosphatase 2C 66 | |  |
| Q99090 | | Light-inducible protein CPRF2 | |  |
| Q7XLR1 | | Probable aquaporin PIP2-6 | |  |
| Q84VY5 | | Probable SAL4 phosphatase | |  |
| Q8H4S6 | | Probable protein phosphatase 2C 64 | |  |
| Q8GY87 | | Probable ubiquitin-conjugating enzyme E2 26 | |  |
| Q8L7M1 | | Probable beta-1,3-galactosyltransferase 14 | |  |
| Q8LPT1 | | ABC transporter B family member 6 | |  |
| Q8RWX7 | | Protein indeterminate-domain 6, chloroplastic | |  |
| Q8RY73 | | Decapping nuclease DXO homolog, chloroplastic | |  |
| Q93VR3 | | GDP-mannose 3,5-epimerase | |  |
| Q94K05 | | T-complex protein 1 subunit theta | |  |
| Q9AV50 | | Double-stranded RNA-binding protein 6 | |  |
| Q9CAR4 | | Probable WRKY transcription factor 36 | |  |
| Q9FGL5 | | Receptor protein-tyrosine kinase CEPR1 | |  |
| Q9FVW3 | | Putative methylesterase 14, chloroplastic | |  |
| Q9LDZ0 | | Heat shock 70 kDa protein 10, mitochondrial | |  |
| Q9LEB4 | | Polyadenylate-binding protein RBP45 | |  |
| Q9SJM3 | | Serine/threonine-protein kinase KIPK2 | |  |
| Q9LFS8 | | Structural maintenance of chromosomes protein 5 | |  |
| Q9LUR0 | | Condensin-2 complex subunit H2 | |  |
| Q9ZRD6 | | VAMP-like protein YKT61 | |  |
| Q9LVQ7 | | Zinc finger protein ENHYDROUS | |  |
| Q9M2H2 | | MATH domain and coiled-coil domain-containing protein At3g58440 | |  |
| Q9S736 | | Protein OBERON 1 | |  |
| Q9SMM0 | | Phosphoglucomutase, chloroplastic | |  |
| Q9SHS7 | | Serine/threonine-protein phosphatase BSL3 | |  |
| Q9XI91 | | Eukaryotic translation initiation factor 5A-1 | |  |
| Q9ZR72 | | ABC transporter B family member 1 | |  |
| Q9ZRE2 | | Ras-related protein RABD1 | |  |
| Q9THZ2 | | Photosystem II reaction center protein L | |  |
| Q6Z4U4 | | LRR receptor kinase BAK1 | |  |
| Q6VAF9 | | Tubulin alpha-4 chain | |  |
| Q43473 | | Tubulin alpha-1 chain | |  |
| Q42368 | | Pyruvate, phosphate dikinase 2 | |  |
| P27793 | | Chorismate synthase, chloroplastic | |  |
| Q41741 | | Eukaryotic initiation factor 4A | |  |
| Q852Q2 | | Serine/threonine protein kinase OSK1 | |  |
| Q07473 | | Chlorophyll a-b binding protein CP29.1, chloroplastic | |  |
| Q0WNJ6 | | Clathrin heavy chain 1 | |  |
| Q5JK68 | | Cyclin-dependent kinase C-2 | |  |
| Q93W28 | | Uncharacterized protein At4g15545 | |  |
| Q93Y37 | | Mannosyl-oligosaccharide 1,2-alpha-mannosidase MNS3 | |  |
| Q94A06 | | Mitogen-activated protein kinase kinase 1 | |  |
| Q94A43 | | BES1/BZR1 homolog protein 2 | |  |
| Q9M2A0 | | ATPase 8, plasma membrane-type | |  |
| F4IGL2 | | Kinesin-like protein KIN-7E | |  |
| P13911 | | DNA-directed RNA polymerase subunit alpha | |  |
| P17770 | | Aromatic-L-amino-acid decarboxylase | |  |
| P51139 | | Glycogen synthase kinase-3 homolog MsK-3 | |  |
| Q07176 | | Mitogen-activated protein kinase homolog MMK1 | |  |
| Q5Z7J0 | | Shaggy-related protein kinase GSK4 | |  |
| Q5Z859 | | Mitogen-activated protein kinase 4 | |  |
| Q8L3X8 | | Serine/arginine-rich SC35-like splicing factor SCL30 | |  |
| Q8RWH3 | | Dual specificity protein kinase YAK1 homolog | |  |
| Q8VY00 | | Pre-mRNA-splicing factor ATP-dependent RNA helicase DEAH1 | |  |
| Q93YN4 | | Mitochondrial fission protein ELM1 | |  |
| Q9FLD5 | | AAA-ATPase ASD, mitochondrial | |  |
| Q9M5P5 | | Probable GTP diphosphokinase RSH3, chloroplastic | |  |
|  | |  | |  |
| **MS/MS Count^b^** | **Fold change^c^** | | **Modified sequence^d^** | |
| 5 | 0.97 | | LLQDS(0.842)LGGNS(0.158)R | |
| 19 | 0.37 | | MAPPPS(1)LAPDR | |
| 2 | -1.18 | | IIRS(1)PEPEVK | |
| 7 | 1.61 | | NRVES(1)WKEK | |
| 4 | -0.24 | | S(1)PDPLEEQR | |
| 7 | 0.53 | | SKS(0.995)YMDS(0.005)KNR | |
| 1 | 1.68 | | GNHPT(0.468)S(0.532)DAFDS(1)T(1)VPVR | |
| 17 | 0.43 | | S(0.001)MS(0.999)FVGTHEYLAPEIIK | |
| 2 | 1.55 | | VLVNIEQQS(1)PDIAQGVHGHFTK | |
| 4 | 1.01 | | LQNLKIMCVRS(1)T(1)GVR | |
| 10 | -0.94 | | S(0.021)YS(0.947)T(0.032)NLAFTPPEYMR | |
| 6 | 0.54 | | RHS(1)FGEDELAYLPDR | |
| 18 | -1.34 | | T(0.006)S(0.006)S(0.987)APPNLDEQKR | |
| 6 | 1.07 | | LDEEENT(0.001)HIS(0.961)T(0.039)R | |
| 3 | 0.67 | | AINS(1)DVDPKS(1)QS(1)PPR | |
| 4 | 0.17 | | S(0.023)YS(0.939)T(0.038)NLAFTPPEYLR | |
| 4 | 0.68 | | S(0.013)FS(0.987)HELNSK | |
| 3 | 0.55 | | KNS(1)EPFMNSLSR | |
| 3 | 0.21 | | NLRKSMEEYICS(1)DPK | |
| 3 | -0.53 | | DQELS(1)QKQNEIEK | |
| 2 | -0.29 | | S(0.988)LRDT(0.986)KPS(0.023)LS(0.002)NLR | |
| 14 | 0.97 | | EES(1)DDDMGFSLFD | |
| 25 | 0.23 | | VEEKEES(1)DEDMGFSLFD | |
| 3 | 0.40 | | T(0.081)RVS(0.096)LS(0.913)S(0.908)LS(0.001)K | |
| 4 | 0.95 | | YGAGIGPGVYDIHS(1)PR | |
| 3 | 0.43 | | RPLQVT(0.004)AS(0.993)PMS(0.002)EFER | |
| 4 | 1.28 | | LVNS(0.035)S(0.965)FADLQKPQVELDGK | |
| 11 | 0.90 | | SMSFS(1)PDRVR | |
| 26 | 0.08 | | AS(0.159)GS(0.841)PPVPVMHS(0.999)PPRPVT(0.001)VK | |
| 18 | 0.85 | | RPFPPPS(1)PAK | |
| 8 | -0.10 | | VAS(0.007)S(0.032)GS(0.961)PWYGPDR | |
| 10 | -0.15 | | SVS(0.002)S(0.01)GS(0.988)PWYGPDR | |
| 2 | 0.05 | | QAS(1)QVVLNDR | |
| 3 | 0.11 | | QLLLHPES(0.21)DDS(0.784)AQLS(0.006)QIETEK | |
| 4 | 0.26 | | TVDGPS(0.17)S(0.83)KDWR | |
| 6 | -0.80 | | PQTET(0.006)KAS(0.993)VGFK | |
| 1 | 1.07 | | NS(0.001)VKNT(0.999)VS(0.999)QVAK | |
| 11 | -0.21 | | EIVT(0.006)S(0.994)PGPEK | |
| 3 | 0.86 | | S(1)RLS(1)AASKPSIAA | |
| 8 | 0.49 | | SSGGLS(1)DDEIDK | |
| 5 | 0.81 | | HGS(0.989)Y(0.011)DKLDDDGLAPPGTR | |
| 5 | 2.28 | | NVIHGS(0.952)DS(0.046)VES(0.002)AR | |
| 3 | -1.22 | | S(0.006)S(0.006)LLHS(0.986)RPKS(0.763)T(0.237)VGT(0.003)PAYIAPEVLSR | |
| 2 | 1.91 | | NFRPDS(1)VVGEGGFGCVFK | |
| 62 | 0.67 | | VEEKEES(1)DDDMGFSLFD | |
| 6 | -1.17 | | KEEPAEES(1)DDDMGFSLFD | |
| 6 | -2.64 | | YHGHS(0.998)MS(0.002)DPGSTYR | |
| 1 | 0.02 | | REIQIMHHLS(1)GQK | |
| 3 | -0.64 | | S(1)DEEHHFESK | |
| 3 | 1.61 | | ASGAFILT(0.213)AS(0.787)HNPGGPNEDFGIK | |
| 15 | -0.50 | | S(1)DLEAPLRPK | |
| 2 | -1.94 | | S(1)AAGTPEWMAPEVLR | |
| 9 | 0.05 | | RLS(1)HLDS(1)IPHT(1)PR | |
| 1 | 1.51 | | LGS(0.957)RLT(0.041)ET(0.002)EVK | |
| 9 | 1.82 | | NLSQMLS(1)QK | |
| 20 | 0.42 | | SMTGEQIQAPS(0.061)S(0.939)PR | |
| 2 | -0.63 | | GHYLNATAGT(0.014)S(0.986)EEMMKR | |
| 1 | 0.69 | | IEKNNS(1)NVS(1)PPR | |
| 7 | -0.97 | | S(0.003)YS(0.972)T(0.026)NLAYTPPEYLR | |
| 2 | 0.23 | | AYLFPES(1)PAR | |
| 6 | 2.15 | | LGANKFS(1)ER | |
| 1 | 1.56 | | GLAY(0.007)DIS(0.992)DDQQDITR | |
| 2 | -1.09 | | VEQLT(0.148)GLS(0.852)GEGR | |
| 67 | 0.99 | | AVGLPTEDDMGNS(1)EVGHNALGAGR | |
| 1 | 0.32 | | GFVPFVPGS(0.797)PT(0.203)ER | |
| 1 | -0.01 | | GKQVLDS(1)GWLAAR | |
| 26 | 0.84 | | S(1)MAQYPEAR | |
| 2 | 1.23 | | S(0.998)NAAS(0.002)KAS(0.001)NIVK | |
| 1 | 0.73 | | ILDVS(1)GNKLR | |
| 2 | 0.62 | | VNS(1)LLNLPR | |
| 20 | 0.22 | | VAS(1)LEHLQKR | |
| 3 | 0.20 | | ALGS(0.013)FRS(0.987)NPS(1)N | |
| 1 | 0.79 | | AIRES(1)IEEEMLLS(0.5)ET(0.5)QLK | |
| 10 | 0.59 | | VNS(1)LVQLPR | |
| 4 | 0.45 | | S(0.105)S(0.126)IS(0.792)IMRS(0.976)QS(0.001)MK | |
| 2 | 0.51 | | T(0.008)KS(0.992)EEKMAQLR | |
| 6 | 0.56 | | RQDS(1)FEMR | |
| 5 | 0.51 | | RRNQPGNPNPDAEVIALS(1)PK | |
| 8 | 0.57 | | DLFGS(1)DNEEYTK | |
| 3 | 0.49 | | VVGTQAPVQLGS(1)LR | |
| 1 | 1.38 | | GTVLIHS(1)AEQLENYAK | |
| 1 | 0.37 | | KQAEKNAAS(1)AAWS(1)S(1)LR | |
| 1 | 0.38 | | S(0.978)ALGFGFQIQS(0.604)Y(0.553)EAS(0.865)K | |
| 2 | -0.06 | | KLWS(0.959)QS(0.959)NKDS(0.076)AS(0.006)EDK | |
| 25 | 0.07 | | TLS(1)DPFSNGK | |
| 7 | 0.27 | | SSGGLS(1)EDDIQK | |
| 13 | 0.48 | | LSWGRS(0.997)PS(0.003)SK | |
| 8 | 0.55 | | SNS(0.999)FVGTHEYLAPEIIK | |
| 1 | 1.88 | | LNLVIGPNGS(1)GK | |
| 2 | 3.60 | | NS(0.998)AHRGS(0.001)S(0.001)VRK | |
| 3 | 0.97 | | GEKLDS(1)LVEK | |
| 15 | 0.59 | | NLPGMPDPDAEVIALS(1)PK | |
| 3 | 1.18 | | S(1)LDLKMQDK | |
| 1 | -0.11 | | S(0.999)DLT(0.001)S(0.618)KT(0.382)LVR | |
| 6 | 2.16 | | ANGGFIMS(0.022)AS(0.978)HNPGGPEYDWGIK | |
| 15 | -0.15 | | QLS(1)IDQFENEGRR | |
| 1 | 0.97 | | SDEEHHFES(0.236)S(0.76)DAGAS(0.004)K | |
| 1 | 0.45 | | NS(0.293)S(0.828)Y(0.031)GRS(0.828)PY(0.001)S(0.018)R | |
| 4 | 0.43 | | S(1)NEYDYLFK | |
| 22 | -1.15 | | T(0.994)QS(0.006)NPNEQNVELNR | |
| 10 | -0.72 | | LMDYKDT(1)HVT(1)T(1)AVR | |
| 13 | 2.23 | | TIQFVDWCPT(1)GFK | |
| 12 | 0.69 | | TVQFVDWCPT(1)GFK | |
| 22 | -2.05 | | GGMT(0.998)S(0.002)HAAVVAR | |
| 1 | -0.33 | | IAFKPT(0.019)S(0.072)T(0.909)IGK | |
| 27 | 0.65 | | VHACVGGT(0.968)S(0.032)VR | |
| 19 | 0.42 | | DGHFLKT(0.99)S(0.01)CGSPNYAAPEVISGK | |
| 10 | -3.70 | | NLAGDVIGT(1)R | |
| 4 | 0.28 | | RPIT(0.986)ADS(0.014)ALMNPNSR | |
| 39 | 0.42 | | SFSSDHNGNLT(1)NR | |
| 3 | 0.04 | | LT(1)PPGS(1)PPILS(0.029)AS(0.314)GT(0.657)PK | |
| 2 | 1.00 | | IS(1)QKGQVNLFET(1)T(1)IR | |
| 3 | -0.27 | | FLTQS(0.006)GT(0.994)FK | |
| 8 | 2.35 | | IS(0.209)NS(0.791)APVT(1)PPLS(0.222)S(0.574)PT(0.126)S(0.078)R | |
| 12 | -0.50 | | GLDIDT(1)IQQHYT(1)V | |
| 2 | 2.09 | | LS(0.021)NGRQGHINY(0.975)RDS(0.003)K | |
| 4 | -0.70 | | S(0.123)NLY(0.933)GT(0.944)REAS(1)ICFK | |
| 1 | 0.79 | | LVCY(0.94)GS(0.126)DQT(0.348)HT(0.585)MFPK | |
| 25 | 1.10 | | VLVKGEPNIS(0.045)Y(0.954)ICS(0.001)R | |
| 27 | 2.63 | | VTSETDFMT(0.002)EY(0.998)VVT(0.001)R | |
| 1 | 0.82 | | GEANIS(0.204)Y(0.784)ICS(0.012)R | |
| 3 | 1.80 | | GQFMT(0.999)EY(0.992)VVT(0.009)R | |
| 8 | 0.37 | | Y(0.007)RS(0.689)RS(0.769)Y(0.847)S(0.688)PAPR | |
| 7 | 0.46 | | TVYS(0.024)Y(0.976)IQSR | |
| 1 | 0.42 | | KELY(1)DLVK | |
| 4 | -0.02 | | IT(0.043)LS(0.964)Y(0.973)KT(0.02)PAK | |
| 2 | 0.76 | | KLY(0.075)S(0.82)NNPS(0.103)QNWS(0.046)GY(0.979)KQT(0.977)K | |
| 2 | 0.27 | | EGISY(0.881)HVVS(0.119)GR | |

a "Protein ID" is determined according to the Uniprot Viridiplantae databasebcde; b “MS/MS Count” means the number of matched phosphopeptides; c “Fold change” indicates log2 fold change of identified proteins in UV-B group compared control group; d “Modified sequence” indicates the amino acid sequence corresponding to the phosphorylated protein.
