# Supplementary material for: Phosphoproteomics Reveals Regulation of Secondary Metabolites in Mahonia bealei Exposed to Ultraviolet-B Radiation
Source: Front Plant Sci. 2022 Jan 11;12:794906. doi: 10.3389/fpls.2021.794906 (PMC8787227; doi:10.3389/fpls.2021.794906)
Supplement: Supplementary file 10 [file Table_3.DOCX]

**Table S3.** List of primers used for qRT-PCR experiments.

| Gene name | Abbreviation | Primer（5’to 3’） |
| --- | --- | --- |
| actin | *act* | GGAGCTGAGAGATTCCGTTG |
|  |  | GAATTCCTGCAGCTTCCATC |
| citrate synthase | *acla* | AAGCGTGGCAAGAGTGGT |
|  |  | AAGCCGATCCGAGACAAC |
| Cytosolic isocitrate dehydrogenase | *cicdh* | GCACAAGGATTCGGGTCA |
|  |  | GTCCACGAGTCCAAGCAA |
| Early light-induced protein 2 | *elip* | GGTGTCGGGTGGTTTCTA |
|  |  | CCTCTGCCCTTATTCCCT |
| Chlorophyll a-b binding protein 4.1 | *lhcb* | TTCAGGTCGGTTCACAGC |
|  |  | CCATCAAGCCAGTCAGGAG |
| 2-oxoglutarate dehydrogenase E1/E2 component | *odha* | CTCCCTGACTGCTAACCCG |
|  |  | TGCCGTCTACGCCCTTGT |
| Pyruvate dehydrogenase E1 component subunit alpha | *osj* | GGCGACTATGTGCCAGGAT |
|  |  | GGTGCGGTAAGTGCTCCCT |
| 2,3-bisphosphoglycerate-independent phosphoglycerate mutase | *pgm* | ATAGTTGACGGTGATGCT |
|  |  | CAAGGTAACGGTTAGGAA |
| (S)-tetrahydroprotoberberine oxidase | *stox* | AGAAGGTGCCACTGCTCT |
|  |  | TTGGACTGATGATTACGG |
| S-adenosylmethionine synthase 1 | *sam* | ATACCTACGGTGGATGGG |
|  |  | AAAGACAGACAATGGCTCA |
